# ML-Based Framework for Urban Thermal Comfort Measurement and Climate Adaptation: Case Study of Barcelona

**DOI:** 10.3390/s26206561

**Published:** 2026-10-16

**Authors:** Vittoria Cipollone, Serena Serroni, Nicolas Cecconi, Edoardo Federici, Nicole Morresi, Gerard Mor, Gian Marco Revel

**Affiliations:** 1 Department of Industrial Engineering and Mathematical Sciences, Università Politecnica delle Marche, Via Brecce Bianche 12, 60131 Ancona, Italy; s.serroni@pm.univpm.it (S.S.); s1115032@studenti.univpm.it (N.C.); s1120129@studenti.univpm.it (E.F.); n.morresi@pm.univpm.it (N.M.); gm.revel@univpm.it (G.M.R.); 2 Department of Engineering and Science, Universitas Mercatorum, 00143 Rome, Italy; nicole.morresi@unimercatorum.it; 3 BEE Group, Centre Internacional de Mètodes Numèrics en Enginyeria (CIMNE), 25003, Lledia, Spain; gmor@cimne.upc.edu

**Keywords:** outdoor thermal comfort, machine learning, Universal Thermal Climate Index, mean radiant temperature, social vulnerability, urban resilience, climate change

## Abstract

Urban heat exposure is one of the most critical and rapidly intensifying impacts of climate change in metropolitan areas. Reliable measurement of outdoor thermal comfort is therefore essential not only for long-term climate-adaptation planning but also for real-time heat-risk management. However, conventional approaches for measuring the Universal Thermal Climate Index (UTCI) rely either on computationally demanding radiative models or on dense in situ monitoring networks, both of which limit scalability and operational deployment at city scale. This study introduces an innovative data-driven framework for urban-scale measurement of mean radiant temperature (t_r_) and UTCI using exclusively open-access reanalysis and real-time meteorological data. A supervised Multi-Layer Perceptron (MLP) model is trained to estimate t_r_ without requiring detailed urban morphology inputs or demanding radiometric instrumentation. The framework achieves high predictive performance (R^2^ = 0.99; RMSE = 1.4 °C for t_r_) and computational efficiency suitable for real-time applications. Another key innovation of this study lies in the comfort–vulnerability integration. The estimated outdoor thermal comfort (i.e., UTCI) is combined with socio-demographic vulnerability measurements within a unified Urban Heat Risk Index (UHRI). Applied to the Besòs district in Barcelona, the framework identifies hotspots where thermal stress and social vulnerability converge, supporting urban resilience and adaptation strategies. A Monte Carlo sensitivity and uncertainty-propagation analysis further showed limited error propagation across the framework, with standard uncertainties of 0.88 °C for MLP-based t_r_, 0.67 °C for UTCI, and 0.017 for UHRI.

## 1. Introduction

Cities are increasingly exposed to the impacts of climate change, with urban heat stress emerging as one of the most critical challenges for contemporary urban environments. Climate change and rapid urbanization have profoundly altered thermal conditions in cities, leading to a marked increase in the intensity and frequency of extreme heat events [1,2]. According to the Intergovernmental Panel on Climate Change (IPCC), global surface temperatures have already risen by approximately 1.1 °C above pre-industrial levels, and continued greenhouse gas emissions are projected to drive further warming throughout the 21st century [3]. Under low-emission scenarios (e.g., SSP1-1.9), warming may be constrained to around 1.4–1.8 °C by 2081 or 2100 compared to pre-industrial levels. However, under higher emission trajectories (e.g., SSP2-4.5, SSP3-7.0, SSP5-8.5), global mean temperatures could reach values between 2.7 and 4.4 °C or more by the end of the century [3]. These increments are expected to amplify the frequency, duration, and severity of heat extremes, with urban populations particularly at risk. At the urban scale, the impacts of global warming are further intensified by local climatic modifiers, most notably the urban heat island (UHI) effect. Dense built surfaces, limited vegetation cover, and restricted ventilation enhance heat storage and re-radiation, resulting in systematically higher temperatures in cities compared to their rural surroundings [1]. The combined effect of large-scale climate warming and local UHI amplification makes urban areas particularly vulnerable to extreme heat. Mediterranean and southern European cities represent particularly relevant contexts for urban heat assessment, as recent decades have been characterized by increasingly frequent and intense heat events, with important consequences for outdoor thermal comfort, heat-related health risks, and cooling demand [4,5]. However, severe thermal stress is not confined to Mediterranean climates, and other regions, including hot-arid, equatorial, and hot-humid environments, may experience different and potentially more challenging combinations of air temperature, solar radiation, and humidity. Within this context, the assessment of outdoor thermal comfort has become a central component of climate-responsive urban planning and public health strategies. Beyond indoor environments, outdoor thermal comfort plays a crucial role in shaping urban livability, influencing human well-being, mobility patterns, and social interaction in public spaces [6,7]. Reliable and robust quantification of outdoor thermal comfort is therefore essential to support both long-term mitigation and adaptation measures in the built environment, as well as short-term operational management during extreme heat events. Outdoor thermal comfort is commonly evaluated through bioclimatic indices such as the Physiological Equivalent Temperature (PET) and the Universal Thermal Climate Index (UTCI) [6]. In the present study, the UTCI has been selected since it provides a widely established measure of outdoor thermal stress based on four environmental variables: air temperature (T_a_), mean radiant temperature (t_r_), wind speed (v), and humidity (RH) [6]. UTCI is calculated through an empirically derived regression function that quantifies the response of the human thermophysiological model over a wide range of environmental conditions. Accordingly, UTCI can be expressed as:

*UTCI* = *T_a_* + *f*(*T_a_*,*t_r_*,*v*,*RH*), (1)

where the correction function accounts for the combined effects of radiative, convective, evaporative, and respiratory heat exchanges. In the present study, this operational UTCI formulation was implemented through the pythermalcomfort library [8,9]. The resulting UTCI value represents an equivalent temperature and is interpreted according to standardized thermal-stress categories, reported in Table 1 [10,11].

It should be noted that UTCI relies on standardized assumptions regarding human activity and clothing insulation and therefore is not intended to reproduce individualized physiological responses under all personal or activity conditions. More detailed thermophysiological models, such as THERMODE and JOS-3, can represent transient human thermoregulation with a higher degree of personalization, but require a broader set of inputs (e.g., body size and anthropometric parameters), metabolic activity, clothing characteristics, and detailed environmental boundary conditions. These requirements are outside the scope of this paper, which is designed to derive spatially continuous thermal-stress information from routinely available environmental data. Accordingly, UTCI is retained here as an urban-scale indicator suitable for spatial mapping and real-time assessment rather than for individualized physiological simulation. The abbreviations used in the current work can be found in Table 2.

Among the environmental variables influencing UTCI, t_r_ has been consistently identified as the dominant driver of perceived thermal stress in outdoor urban environments [6,13]. t_r_ is the uniform temperature of an imaginary enclosure that produces the same radiative heat exchange with the human body as the real environment, including shortwave solar and longwave infrared radiation from all directions [6,14]. Rüdisser et al. [12], for instance, showed that the spatial variation in thermal comfort indices within their urban case study was predominantly associated with variations in t_r_. In sun-exposed urban settings, t_r_ can exceed air temperature by several tens of degrees, making its accurate estimation critical for realistic outdoor comfort assessment. However, obtaining spatially and temporally consistent t_r_ data across complex urban environments remains a major methodological challenge. Traditional approaches for tr assessment can be distinguished into field measurement techniques and computational modeling approaches. Field measurements include black-globe thermometry, commonly applied according to ISO 7726 principles, and multi-directional radiometric measurements, which provide direct information on the local radiative environment but require dedicated instrumentation and are generally limited in spatial coverage [12,13,15]. Computational approaches, instead, estimate t_r_ from meteorological and radiative inputs together with information on urban geometry, shading, sky-view factors, and surface characteristics. Tools such as SOLWEIG, RayMan, and ENVI-met belong to this second category, although they differ in model complexity, required input detail, and computational demand [12]. For example, ENVI-met simulations, even for relatively small urban domains and short simulated periods, can require several hours (typically 8–20 h), limiting their applicability in iterative design workflows and large-scale urban analyses. Moreover, t_r_ estimates show errors (e.g., RMSE up to ~13 °C under unshaded conditions), leading to systematic underestimation of outdoor thermal stress [16]. These limitations restrict their applicability for city-scale analyses and continuous monitoring. Alongside direct measurements and physics-based radiative models, empirical approaches (not based on Machine Learning) have also been developed to estimate t_r_ from available meteorological and radiative variables. Their low computational requirements make them attractive for operational applications. However, their reliance on predefined empirical relationships may limit their ability to represent the complex and nonlinear radiative interactions occurring across heterogeneous urban environments [12]. At broader spatial scales, remote-sensing-based approaches provide a complementary perspective by enabling spatially extensive characterization of urban thermal conditions. Recent studies have demonstrated how the urban thermal environment varies seasonally in response to landscape patterns and multiple environmental drivers [17], while global datasets have enabled the assessment of heatwave exposure associated with the urban heat island effect [18]. Such approaches are particularly valuable for identifying large-scale thermal patterns and heat-exposure hotspots. These studies demonstrate the value of remote sensing for characterizing spatial patterns, environmental drivers, and population exposure to urban heat at regional to global scales. However, this type of assessment addresses a different methodological objective from the direct estimation of t_r_ required for UTCI computation and real-time outdoor thermal comfort measurement, which the current paper instead aims to address. Therefore, despite the complementary capabilities of empirical, physics-based, and remote-sensing approaches, achieving computationally efficient and temporally continuous t_r_ estimation at urban scale remains challenging.

To address this, recent research has increasingly turned to data-driven estimation of t_r_ as a key enabler for city-scale outdoor comfort assessment. A rapidly growing body of work applies artificial intelligence (AI) and machine learning (ML) methods, including ANNs/DNNs, random forests, support vector machines, and neuro-fuzzy systems, to learn the strongly nonlinear relationships between readily available predictors (meteorology, solar geometry, and, when accessible, urban-form descriptors) and t_r_, reflecting the broader diffusion of AI techniques in outdoor thermal comfort modeling [6,19,20,21]. Benchmarking studies indicate that AI-based t_r_ estimators can achieve high predictive accuracy (such as root mean squared errors of 3 °C, coefficient of determination of 0.92) when validated against radiation-based references, while markedly reducing computational costs compared to physically based microclimate models [21,22]. Large-scale measurement campaigns further support their operational potential across diverse urban morphologies. For instance, Jia et al. [12] compared multiple t_r_ estimation techniques across hundreds of measurement sites (including net radiometers and weather stations with globe thermometer) and showed that deep neural networks generally outperform traditional regression approaches in heterogeneous outdoor environments, although residual errors persist in dense urban canyons where dynamic shading and multiple reflections dominate radiative exchange. At the same time, the study highlights a persistent drawback for city-scale deployment: high-quality radiative measurements remain costly and complex to collect at high spatial density. Evidence from large outdoor measurement campaigns suggests that deep learning can deliver high accuracy when trained on diverse, feature-rich datasets. Jia et al., for instance, compared multiple approaches across an extensive set of outdoor measurement sites and reported strong predictive skill for a deep neural network, with coefficients of determination on the order of R^2^ ≈ 0.88 and RMSE around 1.9–2.0 °C depending on the city, outperforming more traditional regression formulations [12]. At the same time, their results underline that performance is not uniform across urban conditions: errors can increase substantially in dense, radiatively complex environments, with site-specific deviations reaching approximately 5 °C in the most challenging locations, consistent with unresolved effects of rapidly changing shading and multiple reflections. In addition, the study explicitly notes that the evaluation is limited to subtropical contexts (Hong Kong and Singapore), indicating that generalization across climates and urban fabrics remains an open issue without broader cross-city validation [12]. Parallel research has demonstrated that ML can emulate numerically simulated radiative environments with dramatic gains in speed, but often by relying on simulation-derived reference t_r_ rather than extensive radiometric ground truth. Xie et al. [23] trained an optimized multi-layer neural network for t_r_ distribution prediction and reported very low errors (e.g., RMSE ≈ 0.49 °C and MAPE ≈ 0.40%) together with computation-time reductions from days to minutes compared with numerical modeling. However, their workflow makes clear that t_r_ values used for training and validation are obtained from simulation-based approaches and the case study is built on simplified, controlled urban configurations [12]. As a consequence, while these studies convincingly show that ML can reproduce simulated t_r_ fields efficiently, the extent to which the learned relationships transfer to real cities, where radiative regimes are shaped by irregular morphologies, heterogeneous materials, vegetation, and highly dynamic sun–shade patterns, may depend strongly on the representativeness of the simulation setup and the realism of the reference data. Operational evidence also exists in closely monitored built environments, where ML-based t_r_ estimation has been successfully integrated into real-time decision systems. Park et al. [22] report that regression trees and ANN models can estimate t_r_ with strong agreement against globe-based measurements in a full-scale residential indoor field deployment, achieving RMSE on the order of 1.3–1.5 °C, and demonstrate downstream benefits when used within comfort-driven HVAC control. Taken together, these studies indicate that the performance of ML-based t_r_ estimation should be interpreted in relation to the microclimatic conditions represented during model development and validation. In particular, the ranges of air temperature, wind speed, solar irradiance, and radiative conditions define the operational domain within which model performance can be more reliably assessed. Previous studies have shown that t_r_ estimation errors may increase under specific radiative conditions, such as sunlit environments and low solar elevations [12]. This is particularly relevant because uncertainty in t_r_ can propagate non-linearly through thermal-stress calculations, affecting downstream indicators and allowable exposure times [24]. Recent literature therefore emphasizes the importance of considering both the environmental domain of model application and the propagation of input uncertainty through thermal-stress assessment frameworks [15].

Nevertheless, the same work is explicitly framed around indoor control applications and highlights that earlier evaluations were conducted in mock-up and simulation-based environments, which differ fundamentally from outdoor urban conditions where direct solar exposure, complex shading, and radiative trapping dominate t_r_ variability [22]. Taken together, the current literature demonstrates that AI can estimate t_r_ with high accuracy and markedly reduced computation time, but it also points to practical constraints that are directly relevant for metropolitan-scale outdoor comfort monitoring: the logistical burden and climate specificity of large measurement campaigns [12], the reliance on simulation-derived labels and simplified geometries in many fast emulation approaches [22], and the fact that some of the strongest operational demonstrations to date originate from indoor or controlled settings rather than spatially heterogeneous outdoor urban environments [22]. These elements motivate approaches that combine computational efficiency with robust validation and that can leverage open, spatially consistent data sources to deliver transferable, real-time t_r_ estimates at the city scale.

The Heat Vulnerability Index (HVI) has emerged as a widely used framework to quantify susceptibility to heat-related impacts by integrating exposure, sensitivity, and adaptive capacity into a composite indicator [7,25]. It is multidimensional, and it is generally measured through combinations of demographic, socioeconomic, health-related, housing, and adaptive-capacity indicators [25,26]. Based on the scope and spatial scale of the study, they can include population age structure, income and deprivation, housing quality and thermal performance, social isolation, energy poverty, and access to cooling resources or services. Hence, the composition of the vulnerability indices changes in accordance with the specific context, spatial resolution, and data availability [20,21,27]. While multidimensional indicator systems provide a more comprehensive representation of heat vulnerability, their implementation at urban scale may be constrained by the spatial availability and comparability of the required socioeconomic and building-related datasets. Numerous studies have shown that incorporating bioclimatic indices such as UTCI into vulnerability frameworks enhances the spatial accuracy of heat-risk identification, particularly in heterogeneous urban contexts [26]. Recent works have highlighted the importance of coupling physiologically based thermal indices with socio-demographic indicators to capture intra-urban disparities in heat risk. Szgradi et al. [26] demonstrated that UTCI-based exposure provides a more robust representation of heat stress than air temperature alone when integrated into social vulnerability models. Similarly, Dong et al. [14] embedded UTCI within a composite heat-health risk index at urban microscale, revealing contrasting patterns between highly exposed urban cores and socially vulnerable peri-urban areas. These studies underscore the need for integrated approaches that combine accurate thermal comfort assessment with vulnerability analysis to support targeted heat-mitigation strategies. Within this context, the present study adopts a modular implementation of the vulnerability component, starting from a single spatially consistent demographic indicator and allowing additional vulnerability dimensions to be progressively incorporated as suitable data become available.

Despite these advances, there remains a lack of scalable frameworks capable of delivering urban-level thermal comfort information in real-time while relying exclusively on open-access data sources and remaining transferable across different urban contexts. In particular, few studies have integrated ML-based t_r_ estimation, UTCI computation, and socio-demographic vulnerability assessment within a unified operational framework suitable for both strategic urban planning and real-time heatwave management.

Addressing these gaps, the present study introduces a supervised ML framework for urban-scale and operational measurement of t_r_ using exclusively open-access reanalysis products from the Copernicus Climate Data Store ERA5, ERA5 Land, and ERA HEAT. A Multi-Layer Perceptron (MLP) is trained on thermodynamic, radiative, and dynamic predictors, including near-surface atmospheric variables and solar geometry features, to reproduce t_r_ accurately without requiring detailed urban morphology or dense radiometric monitoring. The resulting t_r_ fields are then used to compute UTCI, enabling spatially continuous and real-time measurement of outdoor thermal stress across metropolitan areas. Another key innovation is the integration of UTCI with socio-demographic vulnerability indicators within a unified Urban Heat Risk Index (UHRI), supporting robust identification of risk hotspots and delivering actionable outputs for both long-term climate-adaptation planning and real-time heatwave preparedness and response. The proposed technology is applied to a real urban case study: the city of Barcelona. This work is developed within the framework of the Horizon Europe project CLIMRES (Grant Agreement No. 101147777), which aims to enhance the resilience of the built environment to climate change and extreme events through the integration of advanced data-driven methodologies, AI-based tools, and multi-scale risk assessment approaches. The present study contributes to the project by providing a scalable and operational methodology for the assessment of heat-related risk, enabling the characterization of thermal stress and its integration with socio-demographic vulnerability to support urban resilience planning.

The remainder of the paper is structured as follows: Section 2 “Materials and Methods” describes the methodology proposed in this paper, Section 3 outlines the outcomes of the manuscript, while Section 4 discusses the findings and concludes the study with limitations and further directions.

## 2. Materials and Methods

This study implements a data-driven methodology for the measurement of t_r_ and the subsequent computation of the UTCI in urban environments. The methodology combines open-access meteorological data and reanalysis datasets from Copernicus DB (by the European Centre for Medium-Range Weather Forecasts (ECMWF, Reading, UK), data processing for spatial data aggregation and supervised ML models. The methodological workflow is reported in Figure 1, and it aims to support real-time application.

The main steps of the methodology proposed for the real-time measurement of outdoor comfort are:

1. Data acquisition through Copernicus DBs such as ERA5, ERA5-Land, and ERA-HEAT and through the PVlib python (v4.6.1) library. In addition, outdoor environmental data are assessed via the Open-Meteo API for real-time applications, as will be shown in the following chapters;
2. Data Processing focused on spatial and temporal harmonization (through techniques such as bilinear regridding) and de-accumulation procedures to obtain instantaneous measures of some variables such as solar radiation;
3. Feature extraction and ML input dataset creation where a correlation-based approach is implemented according to the literature [28], with the aim of designing an input dataset where features correlate with the target value (i.e., t_r_) and do not correlate with each other (low intercorrelation);
4. ML models’ implementation to measure t_r_. Random Forest, Gradient Boosting and Multi-Layer Perceptron models have been trained, validated and tested;
5. UTCI computation and social vulnerability integration (comfort–vulnerability integration) through an empirical formula where UTCI measurement and the vulnerability factor percentage of people over 65 are mathematically merged;
6. Real-time UTCI measurement based on Open-Meteo API for real-time meteorological data (i.e., air temperature, relative humidity) and Copernicus DB data for quasi-static variables such as the Leaf Area Index or Albedo.

### 2.1. Copernicus ERA5 Reanalysis Datasets

ERA5 is the fifth-generation global atmospheric reanalysis developed by the European Centre for Medium-Range Weather Forecasts (ECMWF) within the Copernicus Climate Change Service. Reanalysis products combine numerical weather prediction models with observations to provide spatially and temporally consistent estimates of atmospheric conditions at the global scale [21]. Compared with conventional meteorological station networks, which provide measurements at discrete locations and may therefore offer limited spatial coverage, ERA5 products provide spatially continuous meteorological information. Previous research has demonstrated the suitability of ERA5 data as an alternative meteorological input for machine learning applications when station observations are spatially limited [22]. ERA5-Land further enhances the representation of land surface conditions by providing meteorological and land surface variables at higher spatial resolution [23]. In addition, ERA5-HEAT provides globally gridded estimates of human biometeorological variables, including tr and UTCI, derived from ERA5 meteorological fields [29]. ERA5-HEAT was specifically developed to provide spatially and temporally consistent information for large-scale thermal comfort assessment, and its methodological formulation and evaluation are documented in the literature [29]. More broadly, ERA5 and ERA5-Land are extensively used as reference environmental datasets in data-driven and ML applications when spatially continuous observations are required [30,31,32,33]. In the present study, ERA5-HEAT was deliberately selected as the reference source for tr because this choice is consistent with the operational objective of the proposed framework. The aim is to provide spatially distributed and temporally continuous estimates of outdoor thermal comfort and heat-related risk at urban scale, including real-time applications in contexts where extensive in situ radiometric monitoring is not feasible. Building the training dataset exclusively from black-globe or radiometric measurements would require a dense and continuously operating monitoring network over the urban domain, thereby reducing the operational applicability of the proposed approach. ERA5-HEAT therefore provides the historical and spatially complete tr target value required for model development, while the trained MLP can subsequently estimate tr from meteorological, radiative, and environmental predictors supplied by operational data streams (i.e., Open-Meteo API). To explicitly account for the uncertainty associated with the use of reanalysis-derived information, a dedicated Monte Carlo uncertainty and sensitivity analysis was incorporated into the methodology (Section 2.8). In the first stage, the uncertainties associated with the meteorological and environmental input variables were propagated through the MLP model to quantify their influence on the predicted tr and to identify which input uncertainties had the greatest effect on the model output. In a second stage, the uncertainty associated with the predicted tr was propagated through the subsequent thermal-stress calculations, including UTCI and persistence component, up to the final UHRI. This analysis therefore provides a quantitative assessment of how uncertainties introduced at different stages of the modeling chain affect the final outputs.

### 2.2. Data Acquisition and Data Processing

The input dataset for t_r_ estimation was built through a sequential pipeline including data extraction, physical transformation, and spatial–temporal harmonization. ERA5-Land and ERA-HEAT variables were complemented with solar geometry parameters computed with the PVlib Python library. The predictor set was designed to represent the main drivers of outdoor thermal comfort, combining near-surface atmospheric conditions, radiative forcing, and land surface properties. Variables used directly as predictors for t_r_ estimation included air temperature at 2 [m] above the surface (t2m, °C), accumulated shortwave solar radiation (R_acc_, J/m^2^), surface albedo (FAL), Leaf Area Index (LAI), relative humidity (RH,%), wind speed and direction (u and v), total cloud cover, and the solar zenith and solar azimuth angles. The latter were computed using PVlib (v 0.16.1) to explicitly account for the temporal evolution and directional dependence of solar exposure. In addition, dew-point temperature (T_d_,°C) was collected but used indirectly, together with the previously described t2m, to derive relative humidity, as will be described below. Therefore, T_d_ was not included as an independent predictor but contributed indirectly through the estimation of RH.

Preprocessing steps were applied to ensure physical consistency and to match the temporal requirements of the machine learning framework. Three main steps were implemented:

1. First, R_acc_ was converted into instantaneous irradiance (W/m^2^) by differencing consecutive accumulation values and dividing by the hourly time interval (3600 s).

(2) $$R_{\mathrm{inst}}\left(i\right)=\frac{R_{\mathrm{acc}}\left(i\right)-R_{\mathrm{acc}}\left(i-1\right)}{3600};$$

2. Second, outdoor relative humidity (RH, %) was derived from t2m and T_d_ using the Tetens formulation, which computes saturation and actual vapor pressure as exponential functions of temperature and expresses relative humidity as their ratio:

(3) $$e_{s}=6.112\times \exp\left(\frac{17.67\cdot T_{\mathrm{out}}}{T_{\mathrm{out}}+243.5}\right),\;e=6.112\times \exp\left(\frac{17.67\cdot T_{d}}{T_{d}+243.5}\right),\;\mathrm{RH}=100\times \frac{e}{e_{s}}.$$

Once all input variables were processed and temporally aligned, spatial harmonization was required due to differences in the native grid resolution of the input sources. Most features (e.g., ERA5-Land) were available at 0.1° × 0.1°, whereas cloud cover and the target variables (t_r_ and UTCI; ERA5 and ERA-HEAT) were provided on a 0.25° × 0.25° grid. To enable this correspondence between features and targets, all fields were resampled onto a common grid using bilinear interpolation, which estimates target values as a distance-weighted combination of the four neighboring source-grid points [20]. At domain boundaries, where fewer neighbors are available, a nearest-neighbor fallback strategy was applied to ensure complete spatial coverage. In other words, this procedure computes the value of a variable at a target location (*x*,*y*) as a weighted average of the four surrounding points on the source grid:

(4) $$I^{\prime }(x,y)=\alpha I(x^{\prime },y^{\prime })+\beta I(x^{\prime }+1,y^{\prime })+\gamma I(x^{\prime },y^{\prime }+1)+\delta I(x^{\prime }+1,y^{\prime }+1).$$

The weights are defined by the relative distance between the target location and the neighboring grid nodes:

*α* = (1 − Δ*x*)(1 − Δ*y*), *β* = Δ*x*(1 − Δ*y*), *γ* = (1 − Δ*x*)Δ*y*, *δ* = Δ*x*Δ*y*,

with:

$$\Delta x=s_{x}^{-1}\left(x,y\right)-x^{\prime },\;\Delta y=s_{y}^{-1}\left(x,y\right)-y^{\prime }.$$

The resulting dataset, fully harmonized in space and time, was then used as input to the ML pipeline for t_r_ estimation.

### 2.3. Feature Extraction

A correlation-based feature selection (CFS) approach, widely established in the literature [34,35], was adopted during the feature extraction stage. This methodology aims to evaluate both the relationship between each candidate feature and the target variable, used as the label of the ML model, and the intercorrelation among the input features. Specifically, Pearson’s correlation coefficient (R) was computed for all considered variables and summarized in the correlation matrix presented in Figure 2.

This analysis allowed features showing a strong linear association with the target variable to be identified while simultaneously highlighting potential redundancies among the inputs. Particular attention was therefore devoted to minimizing multicollinearity within the input dataset, since the inclusion of highly correlated features may introduce redundant information, increase model complexity, and potentially increase the risk of overfitting during model training. Accordingly, features characterized by a meaningful correlation with the target and, at the same time, limited correlation with the other predictors were preferentially retained. Since Pearson’s correlation coefficient ranges from −1 to +1, values approaching ±1 indicate a strong linear relationship, whereas values close to zero indicate a weak or negligible linear correlation. As shown in Figure 2, several strong inter-feature correlations were identified. Solar elevation, despite showing a strong positive correlation with t_r_ (R = 0.86), was excluded because of its high correlation with the solar radiation variable (R = 0.85) and because of the perfectly negative correlation with solar zenith (R = −1.00), thus providing largely redundant information. Similarly, the surface solar radiation downwards (ssrd_current, Figure 2) and the surface net solar radiation (ssr_current, Figure 2) exhibited an almost perfect positive correlation; therefore, only the first of the two variables was retained in the final feature set. Vapor pressure was also excluded during the preliminary feature-selection stage: although it exhibited a moderate correlation with t_r_ (R = 0.43), it was strongly associated with temperature and humidity-related variables and was therefore considered to provide limited additional independent information. These choices were made to reduce multicollinearity risk while preserving variables containing complementary information for t_r_ prediction. Overall, the resulting feature set was designed to provide an appropriate balance between predictive information and predictor independence, thereby reducing unnecessary model complexity and the risk of overfitting and improving the robustness and generalization capability of the ML model [21,22]. A complete overview of input features and target variables, including data sources and units, is reported in Table 3.

### 2.4. ML-Based t_r_ Measurement

Once the input dataset had been prepared, the ML pipeline for t_r_ estimation was developed. Consistent with previous studies, three supervised regression algorithms were considered, namely Random Forest (RF), Gradient Boosting Regressor (GBR), and Multi-Layer Perceptron (MLP). The hyperparameter settings used for each model are reported in Table 4 and were selected through a grid search with cross-validation, retaining the configuration that achieved the best validation performance. The chosen methods span distinct regression approaches, including tree-based ensemble learners and a feed-forward neural network, and they allow a rigorous comparison of how effectively nonlinear links between radiative, thermodynamic, and dynamical predictors are represented.

Model development and internal evaluation relied on observations spanning the full year 2023, thereby encompassing all seasons and representing the complete range of meteorological and radiative conditions. The dataset was partitioned into independent training and test sets using a holdout method, with 80 percent of the samples used for training and the remaining 20 percent reserved for testing. This split supports learning and assessment under heterogeneous environmental regimes. Model performance was quantified using four standard regression metrics, namely mean absolute error (MAE), root mean squared error (RMSE), normalized root mean squared error (NRMSE) and the coefficient of determination (R^2^). The corresponding definitions are provided below, where *y_i_* is the measured value, $\tilde{y_{i}}$ is the predicted value, and $\hat{y_{i}}$ is the mean of the measured values.

(5) $$\mathrm{MAE}=\frac{1}{n}\sum_{i=n}^{n}\left|y_{i}-\tilde{y_{i}}\right|,$$

(6) $$\mathrm{RMSE}=\sqrt{\frac{1}{n}\sum_{i=n}^{n}{\left(y_{i}-\tilde{y_{i}}\right)}^{2}},$$

(7) $$\mathrm{NRMSE}=\frac{\mathrm{RMSE}}{y_{\max}-y_{\min}},$$

(8) $$R^{2}=1-\frac{\sum_{i=1}^{n}{\left(y_{i}-\tilde{y_{i}}\right)}^{2}}{\sum_{i=1}^{n}{\left(y_{i}-\hat{y_{i}}\right)}^{2}}.$$

Following model training, an out-of-sample evaluation (test of the trained model) was carried out using the best-performing algorithm identified during training, namely the MLP, as indicated by the validation metrics in Table 5. To measure model generalization while preserving the same spatial domain, the MLP was tested on an independent dataset referring to a different year. In particular, the testing phase used data from August 2024.

### 2.5. UTCI Computation and Social Vulnerability Integration

After estimating t_r_ with the machine learning model, outdoor thermal comfort was quantified by computing the UTCI, which was computed using the open-source `pythermalcomfort`. The UTCI implementation directly uses air temperature, mean radiant temperature, wind speed, and relative humidity as input variables and applies the standard UTCI operational approximation. Internally, the package evaluates the UTCI polynomial approximation using these four environmental inputs, thereby providing a computationally efficient and fully reproducible estimate of UTCI without the need to explicitly execute the underlying thermophysiological model. Because all variables are defined on the reanalysis grid, the resulting UTCI outputs are inherently georeferenced and can be represented as spatially continuous raster fields over the study domain, with each value associated with a specific latitude and longitude. UTCI values were subsequently categorized according to the official ISB UTCI thermal-stress classes [11,36], spanning from no thermal stress to extreme heat stress (Table 1). As an example, a UTCI value ranging from 9 to 26 was categorized as “no heat stress”.

Once UTCI was computed and categorized as a georeferenced indicator of outdoor thermal exposure, the analysis proceeded with the integration of a social vulnerability component. From the literature, vulnerability was quantified as the percentage of residents aged 65 years and older within each administrative unit, derived from official census statistics available at census zone level [37]. It is acknowledged that the proportion of elderly residents does not capture all dimensions of socioeconomic vulnerability. Indeed, vulnerability is a multidimensional concept influenced by several factors, including income level, health conditions, social isolation, and access to cooling systems. Nevertheless, in this initial implementation of the UHRI, the study focused exclusively on the elderly population because older individuals are widely recognized as more susceptible to heat stress and often exhibit a reduced capacity to cope with extreme heat conditions [37]. Furthermore, the selection of this variable was primarily guided by data availability, spatial completeness, and consistency across the study area, which are essential requirements for integrating demographic information with high-resolution climatic fields. Therefore, the percentage of elderly residents was selected as a reliable and spatially consistent indicator that can be systematically integrated with thermal exposure metrics at the metropolitan scale. At the same time, its use should not be interpreted as a comprehensive representation of social vulnerability, but rather as a first implementation of the vulnerability component within the proposed heat-risk framework, which can be progressively extended to include additional dimensions as suitable spatially consistent data become available. In fact, this framework is designed to be modular and extensible, allowing further vulnerability indicators to be incorporated as they become available at comparable spatial resolution.

From an operational standpoint, to enable this integration, the georeferenced variable UTCI has been spatially aligned to administrative or census units. Specifically, UTCI values were aggregated at the polygon level using inverse distance weighting. For each polygon centroid *x*_0_, the aggregated UTCI value was computed as a distance-weighted average of surrounding raster cells:

(9) $$z\left(x_{0}\right)=\frac{\sum_{i}w_{i}\;z_{i}}{\sum_{i}w_{i}},\;w_{i}=\frac{1}{d_{i}^{\;p}},\;p=1.8,$$

where *z_i_* is the UTCI value at raster cell *i*, *d_i_* is the distance between the raster cell center and the polygon centroid, and *p* is the distance decay exponent. In this study, *p* = 1.8 was selected based on empirical testing to balance local sensitivity and spatial smoothing. This aggregation approach is computationally efficient and suitable for repeated processing at metropolitan scale, and it is useful for subsequently computing the UHRI. The spatial aggregation procedure based on building centroids and IDW interpolation is schematically illustrated in Figure 3.

UHRI score was formulated to combine thermal-stress derived from the AI-based outdoor comfort module with the single vulnerability indicator, namely the percentage of residents aged 65 years and older and an additional temporal descriptor of heat stress persistence. In this study, UHRI is developed through a linear combination of three different components: UTCI, persistence and vulnerability. The UTCI is measured through AI and computed for each administrative spatial unit (census-based polygons). The second component (*Persistence*) represents the duration of continuous thermal-stress exposure, measured as the number of consecutive hours during which UTCI exceeds predefined thermal stress thresholds. Depending on the application context, persistence values may also be grouped into categorical ranges to distinguish different levels of prolonged heat exposure [38]. The third component represents demographic vulnerability (i.e., Over65) within each spatial unit. To ensure comparability across administrative units with different population sizes and climatic distributions, each component was normalized prior to aggregation. The resulting risk score for spatial unit *i* was computed as:

*UHRI* = *α UTCI* + *β Persistence* + *γ Over*65. (10)

The weighting coefficients (α = 0.35, β = 0.35 and γ = 0.30) were empirically defined through a feedback-oriented expert evaluation procedure designed specifically for the proposed UHRI framework. Rather than being directly transferred from previous studies, the selected weights reflect a balanced assessment of the relative importance of thermal intensity, persistence of heat stress conditions, and demographic vulnerability within the study context. Existing literature was primarily used as methodological support for adapting an expert-informed weighting strategy, which is a common practice in heat vulnerability and multi-criteria risk assessment frameworks, instead of relying exclusively on statistical or optimization-based approaches [25]. To evaluate the influence of the expert-defined weighting coefficients on the resulting UHRI, a sensitivity analysis was performed using the Saltelli sampling method. Saltelli is a variance-based approach that generates different combinations of the input parameters in a structured manner, allowing the influence of each parameter on the variability of the output to be quantified through Sobol sensitivity indices. In particular, the first-order index S_1_ represents the contribution of an individual parameter to the output variance, the second-order index S_2_ represents the contribution arising from the interaction between pairs of parameters, and the total-order index S_T_ accounts for the overall contribution of a parameter, including both its individual effect and all interaction effects involving that parameter. A base sample size of N = 1024 was adopted, where N represents the number of base samples and D = 3 corresponds to the three uncertain UHRI weighting coefficients. Since second-order sensitivity indices were not estimated, the Saltelli sampling design resulted in (N(D + 2) = 5120) model evaluations. For each generated combination, the UHRI was recalculated using the corresponding weighting configuration. First-order S_1_ and total-order S_T_ Sobol indices were calculated to quantify how variations in each individual weighting coefficient affected the variability of the index. In this way, the analysis provided a quantitative assessment of the sensitivity of the proposed index to the expert-defined weighting scheme and allowed the relative influence of the three coefficients to be identified. Since UHRI is intended to support comparison among different spatial units, it should be interpreted as a relative composite heat-risk indicator rather than as an absolute quantitative estimate of health risk. The resulting UHRI values were then classified into four relative risk categories, considering that the overall range of the indicator varies from 0 to 1, as summarized in Table 6. These categories represent levels of combined thermal exposure, heat stress persistence, and demographic vulnerability within the proposed framework and should not be interpreted as absolute probabilities or quantitative estimates of heat-related health outcomes.

### 2.6. Real-Time Application

The ML-based approach presented above also enables the implementation of an operational workflow for real-time assessment of outdoor thermal comfort at urban scale, addressing key limitations identified in previous studies. While the core contribution of this work lies in the integration of AI-based t_r_ estimation, UTCI computation, and socio-demographic vulnerability within a unified urban heat risk framework, the proposed pipeline additionally supports the real-time application of this measurement procedure through automated data ingestion. The trained and tested MLP model is used to estimate t_r_ at hourly resolution by providing, as input features, the variables listed in Table 7. For real-time operation, the meteorological predictors (Table 7) are acquired via API from the Open-Meteo online weather service. In addition, quasi-static land surface descriptors (Table 7), specifically LAI and FAL (ERA5 Land), are included to represent background surface conditions. Solar geometry variables (i.e., solar zenith and solar azimuth angles) are computed hourly using the PVlib library.

The use of different meteorological data sources between model development and operational deployment introduces an additional source of uncertainty that should be considered when interpreting the real-time t_r_ estimates. The MLP was trained and validated using ERA5-derived meteorological variables, whereas the operational implementation relies on real-time data retrieved from the Open-Meteo API for several atmospheric predictors, including air temperature, relative humidity, wind conditions, solar radiation and cloud cover. This choice was necessary to meet one of the main objectives of the present study, namely the development of a framework capable of providing real-time thermal environment estimates over large spatial domains. ERA5 reanalysis products are particularly suitable for model development and retrospective analysis, but they are not intended to provide the same operational real-time data stream required by the proposed application. For this reason, Open-Meteo was adopted during the operational phase as a practical source of continuously updated meteorological information. This potential source-dependent bias was also quantitatively investigated and was found to be relatively limited for some key predictors. The comparison between the two data sources, conducted for August 2024, showed generally small systematic differences for air temperature and wind speed, with a mean bias of +0.07 °C for air temperature, while dew-point temperature showed a slightly larger offset of −1.15 °C. Solar radiation also exhibited a very small mean bias (+2.15 W/m^2^). Greater discrepancies were observed for low-cloud cover, with a mean bias of +3.15 percentage points, although this variable is also characterized by a broader numerical range and substantial temporal variability. Although the use of different meteorological data sources may introduce potential source-dependent bias or distribution shifts, Open-Meteo remains the most suitable operational option for the objectives of the present study, as it provides continuously updated meteorological information required for real-time application. The hourly t_r_ estimates are then used to derive UTCI using the procedure described previously. The resulting UTCI fields are subsequently aggregated to administrative units and integrated with socio-demographic vulnerability information to generate heat risk metrics by using the UHRI (Equation 9), as outlined in the preceding section. The final outputs consist of georeferenced t_r_ and UTCI measures, updated at hourly intervals. The overall processing chain is organized as a modular pipeline comprising data ingestion, feature preparation, machine learning inference, and index computation, supporting reproducibility and enabling consistent deployment in both offline analyses and real-time applications.

### 2.7. Case Study: Barcelona

The proposed outdoor thermal comfort assessment workflow was deployed in Barcelona, Spain, specifically within the Sant Martì district (41°25′7.54″ N, 2°12′20.05″ E—part of El Besòs y el Maresme zone) as a representative real-world urban case study (Figure 4). The neighborhood covers approximately 1.27 km^2^ and hosts a population of 22.600 inhabitants, resulting in a population density of about 18.000 inhabitants per km^2^. The area is characterized by a compact urban morphology and a residential building stock largely developed between 1959 and 1965 in response to the post-war housing demand. The neighborhood includes nearly 9.800 dwellings, with almost 90% of the housing stock constructed before 1980. Consequently, many buildings exhibit limited thermal performance due to poor envelope insulation and outdated construction technologies. These characteristics make the area particularly suitable for investigating outdoor thermal comfort, as the local population is likely to be highly exposed to urban heat stress, a risk that is expected to intensify with rising temperatures and more frequent heatwave events. To ensure consistency with the spatial support of the reanalysis predictors, the analysis domain was expanded from the district scale to the broader Barcelona metropolitan area, covering both urban and peri-urban grid cells within a Mediterranean climate regime.

Hourly model inputs were extracted for each grid cell and comprised the set of meteorological, radiative, and land surface predictors previously defined. t_r_ was selected as the prediction target and was benchmarked against reanalysis-based estimates provided by Copernicus ERA5 HEAT. The modeling framework described in the previous paragraph was applied using hourly data from the entire year 2023, covering 12 grid cells arranged in a 3 × 4 spatial grid over the Barcelona metropolitan area (latitudes 41.3° N–41.5° N, longitudes 2.0° E–2.3° E, spatial resolution 0.1°). The dataset was randomly divided into training (80%) and validation (20%) subsets, yielding approximately 84.000 and 21.000 hourly observations, respectively. Model performance was then tested using an independent testing dataset collected in August 2024, a period characterized by high radiative loads and recurrent heatwave conditions, thus providing a demand assessment of predictive robustness under severe summer heat stress.

### 2.8. Monte Carlo Uncertainty Analysis

Since the proposed framework combines reanalysis-derived variables, machine learning predictions and subsequently derived thermal-risk indicators, an uncertainty-propagation analysis, as previously mentioned, was performed to quantify how uncertainties introduced at different stages of the modeling chain affect the final outputs. A Monte Carlo approach, based on repeated random sampling according to the GUM framework [39], was adopted to propagate uncertainty first from the meteorological and environmental input variables to the estimated t_r_, and subsequently from t_r_ through UTCI and the persistence component to the final UHRI. This approach has also been applied to uncertainty assessment in AI-based predictive models, as demonstrated by Morresi et al. [40]. A total of 10^6^ Monte Carlo realizations were used for each uncertainty-propagation experiment to characterize the resulting output variability.

The first stage of the analysis focused on the MLP-based t_r_ estimation. For each uncertain input variable, the original value was perturbed at every Monte Carlo iteration by adding a random error consistent with the uncertainty associated with that variable. Assuming a Gaussian distribution, the perturbed value of the i-th input variable at the k-th iteration was calculated as:

(11) $${{X\;}_{i}^{\left(k\right)}=x}_{i}\;+\sigma_{i}z_{i}^{\left(k\right)},$$

where *x_i_* is the original value of the input variable, *σ_i_* is the corresponding standard deviation used to represent its uncertainty, and $z_{i}^{\left(k\right)}$ is a random value drawn from a standard normal distribution. The uncertainty parameters *σ_i_* were derived from ERA5 uncertainty information. The resulting distribution of t_r_ predictions was then analyzed to quantify the variability in model output induced by uncertainty in each individual predictor. This one-at-a-time analysis was used to identify which input uncertainties had the greatest influence on t_r_ estimation. Subsequently, all uncertain input variables were perturbed simultaneously within each Monte Carlo realization. A sensitivity coefficient, c_i_, was also calculated for each input variable as the ratio between the propagated standard uncertainty in t_r_ and the standard uncertainty of the corresponding input variable:

(12) $$c_{i}=\frac{u_{i}\left(t_{r}\right)}{u\left(x_{i}\right)},$$

where *u*(*x_i_*) is the standard uncertainty associated with the i-th input variable and *u_i_*(*T_r_*) is the corresponding propagated standard uncertainty in the predicted t_r_.

In a second stage, the Monte Carlo approach was also applied to investigate how the uncertainty associated with the MLP-predicted t_r_ propagates through the subsequent steps of the thermal-risk assessment, from UTCI to the final UHRI. The prediction errors obtained from the MLP were first characterized in terms of their statistical distribution and used to define the uncertainty associated with the estimated t_r_. For each Monte Carlo realization, UTCI was recalculated using the perturbed t_r_, while air temperature, relative humidity, and wind speed were kept unchanged. This procedure was adopted to specifically isolate the effect of uncertainty in the MLP-estimated t_r_ on the resulting UTCI. To further evaluate how this uncertainty propagates to the final UHRI, the resulting UTCI values were then used to recalculate the persistence component and, subsequently, the UHRI. As previously defined in Equation 10, UTCI contributes to the vulnerability index through two pathways: directly as a normalized component of the index and indirectly through the persistence term, which is itself derived from the temporal evolution of UTCI. Therefore, for each Monte Carlo realization, the perturbed UTCI series was first used to recalculate persistence. The simulated UTCI and persistence values (Eq. 10) were then normalized using the same reference ranges adopted in the baseline calculation and combined with the unchanged normalized over-65 population component to obtain a new UHRI value. The propagated uncertainty was expressed as uncertainty U using a coverage factor k = 2. Sensitivity coefficients were also calculated for the downstream outputs according to the same formulation described above for the first-stage analysis. This procedure allowed the effect of t_r_ prediction uncertainty to be tracked consistently across the entire downstream calculation chain, from t_r_ to UTCI, persistence, and finally UHRI. The Monte Carlo analysis therefore provided a quantitative assessment of the extent to which uncertainty introduced at the t_r_ prediction stage propagates through the thermal-stress and heat-risk indicators.

## 3. Results

### 3.1. t_r_ and UTCI Measurement Results

This section presents the results of the ML-based estimation of t_r_ and the subsequent computation of UTCI. The first set of results concerns t_r_ predictions obtained during model training and internal testing. As summarized in Table 5, the MLP achieved strong agreement with reanalysis-based reference values, with MAE equal to 1.1 °C, RMSE equal to 1.4 °C, NRMSE equal to 1.9% and R^2^ = 0.99. The corresponding performance is also shown in Figure 5.

In particular, Figure 5b reports the relationship between predicted t_r_ and the reference t_r_ extracted from the reanalysis dataset, highlighting the close correspondence across the full range of values. UTCI was then derived using the predicted t_r_ together with t2m, RH, and u_wind_, following the standard procedure described previously. The resulting UTCI fields were evaluated against ERA5 HEAT UTCI reference values. As reported in Table 8 and Figure 6, UTCI derived from the ML outputs achieved an MAE equal to 1.2 °C, an RMSE equal to 1.6 °C, and an R^2^ = 0.99.

To assess model generalization, the trained and validated MLP was subsequently tested on a new dataset collected back in August 2024, which was not included in the training process. As shown in Table 8, when moving from seen to unseen data characterized by different seasonal and meteorological conditions, the performance errors for estimating t_r_ increase moderately (MAE = 1.9 °C, RMSE = 2.6 °C), while the coefficient of determination remains high (R^2^ = 0.99). This behavior reflects a typical outcome: the model retains its explanatory power and the loss in accuracy remains limited, with error magnitudes still within a comparable °C range with respect to training (Figure 7). 

Moreover, the obtained test performance is comparable to, and in some cases better than, values reported in previous ML-based t_r_ estimation studies. The corresponding UTCI values were then calculated using the predicted t_r_ together with air temperature, relative humidity, and wind speed, and were compared with ERA5-HEAT reference values. Interestingly, the UTCI performance did not show the same degradation observed for t_r_. On the contrary, the August 2024 test dataset yielded slightly lower errors, with MAE decreasing from 1.2 °C to 0.9 °C and RMSE from 1.6 °C to 1.2 °C, while R^2^ remained equal to 0.99 (Table 8 and Figure 8).

This result should not necessarily be interpreted as an improvement in the generalization capability of the model, but rather as an indication that the larger t_r_ errors observed during the test period did not propagate proportionally to the derived UTCI. Indeed, UTCI is determined by the combined effect of t_r_, air temperature, relative humidity, and wind speed, and its response to variations in an individual input variable is therefore not linear or constant across all meteorological conditions. The specific range and combination of thermal and meteorological conditions occurring during August 2024 may consequently have limited the influence of t_r_ prediction errors on the final UTCI values. In addition, because both the calculated UTCI and the ERA5-HEAT reference are based on consistent reanalysis-derived meteorological information, part of the high agreement may also reflect the consistency of the underlying input data. The propagation of these uncertainties through the different stages of the methodology is further investigated through the Monte Carlo analyses presented in Section 2.7.

Finally, since the generated outputs are fully georeferenced, spatial maps of the ML-based t_r_ and UTCI were produced (Figure 9, Figure 10 and Figure 11), providing a visual representation of their distribution across the study area. Hourly snapshots at 01:00, 09:00 and 13:00 LST, averaged over August 2024, illustrate the representative diurnal cycle across the Barcelona metropolitan area. At 01:00 (Figure 9), t_r_ and UTCI reach their nocturnal minimum, with slightly higher values along the coast due to the greater thermal inertia of the sea, which releases stored heat more slowly than land surfaces [41]. At 09:00 (Figure 10), increasing solar irradiance raises both indices, with marginally higher values inland, although the inland-coastal contrast remains limited during this transitional phase. At 13:00 (Figure 11), t_r_ and UTCI reach their daily maxima, particularly in inland and central urban areas, where intense solar exposure, radiative trapping, and limited ventilation enhance thermal stress. Coastal areas remain cooler, consistent with the moderating effect of sea breezes on daytime thermal conditions [42].

### 3.2. Comfort–Vulnerability Integration

This section presents the results of the integration between outdoor thermal comfort (i.e., UTCI) and the demographic vulnerability indicator Over65. The resulting UHRI, computed according to Equation (10) and classified into the four predefined score ranges, is shown in Figure 12. The spatial distribution of the UHRI reveals a pronounced gradient across the metropolitan area, with higher index values concentrated in central and semi-central districts. These areas are assigned to the orange and red classes, indicating the occurrence of persistent thermal stress and a higher proportion of residents aged over 65 years. Neighborhoods such as Gràcia, Sants-Montjuïc, Eixample, and Ciutat Vella emerge as potential priority spots according to the components included in the proposed UHRI, consistent with their dense urban fabric and aging population structure [43,44]. In contrast, coastal sectors and areas with greater vegetation cover are predominantly classified within the lower UHRI categories. These spatial patterns are consistent with the mitigating influence of the sea and greener urban environments, which can reduce thermal load through moderated air temperatures, enhanced ventilation, and lower radiative stress. Overall, Figure 12 identifies areas with higher UHRI values, therefore indicating locations where the considered thermal (UTCI) and demographic (Over65) risk components jointly reach comparatively higher levels. In the present study, however, the UHRI should be interpreted as a spatial screening and prioritization index rather than as a quantitatively validated predictor of population-level health outcomes. Nevertheless, the identified areas serve as triggers for preventive interventions and heat-mitigation and adaptation planning.

### 3.3. Real-Time Thermal Comfort Results

The integration of the trained MLP model with real-time meteorological data streams enables the operational generation of real-time t_r_ and UTCI estimates across the study domain. The hourly temporal resolution was retained throughout the workflow, including the UHRI computation, to support identification of critical intra-day exposure windows and accurate quantification of heat stress persistence (Equation (10)). As described in the sensitivity analysis, variations in the weighting coefficients affected the UHRI for different measures. The UTCI weight α showed the greatest influence on the variability of the index (S_1_ = S_T_ = 0.60), followed by the demographic vulnerability weight γ (S_1_ = 0.37), whereas the persistence weight β showed a substantially lower influence (S_1_ = S_T_ = 0.03). This indicates that the UTCI component is the main driver of UHRI variability and, consequently, exerts the strongest influence on the resulting spatial risk patterns and hotspot identification, consistently with the spatial results obtained in the case study. The Monte Carlo analysis presented in the following section further showed that the propagated uncertainty has only a limited effect on the final normalized UHRI. The similarity between the first-order and total-order indices is consistent with the independent treatment of the three weighting coefficients and their additive contribution to the UHRI formulation, resulting in negligible interaction effects among them. This configuration allows scalable and location-independent deployment of the modeling framework. Figure 13 illustrates an example of hourly t_r_ estimates produced in real time for the district under study. The predicted values reproduce the expected daily dynamics of t_r_, characterized by a rapid increase after sunrise, a peak around solar noon, and a gradual decline during the afternoon and evening hours. This behavior confirms the capacity of the model to capture short-term radiative variability under operational conditions. The corresponding UTCI evolution is shown in Figure 13b. UTCI is computed from the estimated t_r_ in combination with forecast air temperature, wind speed, and relative humidity. UTCI reflects the combined thermophysiological effects of multiple atmospheric variables, resulting in a more comprehensive representation of perceived thermal comfort. For operational interpretation, UTCI values are evaluated against commonly adopted warning thresholds (e.g., 32 °C and 38 °C) [45], which denote strong and very strong heat stress (Figure 13). The comparison with these thresholds enables the identification of critical time windows during which exposure may pose significant health risks.

### 3.4. Monte Carlo Uncertainty Analysis Results

The Monte Carlo analysis, based on 10^6^ realizations, quantified the propagation of uncertainty through the different stages of the proposed framework. In the first stage, the one-at-a-time perturbation of the MLP input variables showed that the resulting variability in predicted t_r_ differed considerably among the considered predictors. As reported in Table 9, high-vegetation LAI produced the largest t_r_ standard deviation, followed by relative humidity, low-vegetation LAI, and surface solar radiation.

Conversely, solar zenith and azimuth angles produced only a limited variation in the predicted t_r_. Overall, the uncertainty propagated from each individual input resulted in relatively limited dispersion of the predicted t_r_, with all one-at-a-time mean standard deviations remaining below 0.5 °C. The corresponding sensitivity coefficients quantify the ratio between the propagated standard uncertainty in t_r,_ and the standard uncertainty assigned to each input variable. Since the predictors are expressed in different physical units, these coefficients should be interpreted as variable-specific uncertainty-propagation factors rather than directly compared across all predictors. When all uncertain input variables were perturbed simultaneously, the combined t_r_ propagated standard uncertainty increased to 0.88 °C. This result indicates that, within the uncertainty ranges adopted in the Monte Carlo analysis, the MLP predictions remain relatively stable with respect to perturbations in the input variables. The combined uncertainty is naturally higher than that obtained from the individual perturbations, as it reflects the simultaneous propagation of uncertainty from all considered predictors.

In the second stage, the uncertainty associated with the MLP-predicted t_r_ was propagated through the downstream thermal-risk calculations. Using a coverage factor k = 2, the resulting propagated uncertainties U(y) were 0.67 °C for UTCI, 0.34 h for persistence, and 0.017 for the final UHRI. Sensitivity coefficients were also calculated according to the formulation described in Section 2.7 to quantify the propagation of t_r_ prediction uncertainty to the downstream outputs. The coefficient associated with the propagation from t_r_ to UTCI was 0.26, while that associated with the subsequent propagation from UTCI to persistence was 0.51. A single sensitivity coefficient was not assigned to UHRI because the index depends simultaneously on both the normalized UTCI and persistence components. The results are summarized in Table 10.

Collectively, these results show that uncertainty introduced at the t_r_ prediction stage propagates through the subsequent thermal-risk calculations but progressively decreases along the modeling chain. In particular, the relatively small uncertainty obtained for the final U_UHRI_ = 0.017 indicates that the normalized heat-risk indicator is only moderately affected by uncertainty originating from the MLP-based t_r_ estimation.

## 4. Discussion and Conclusions

This study presented and validated a novel data-driven framework for urban-scale measurement of t_r_ through an ML model, supporting the computation of citizens’ outdoor thermal comfort (i.e., UTCI), and the integration of it with socio-demographic vulnerability to generate an operational UHRI. The proposed methodology is conceived as an urban-scale decision-support framework for municipalities, local authorities, urban planners, and civil-protection services. At its present stage, its most appropriate application is the spatial and temporal screening of thermal comfort and heat-risk conditions, allowing decision makers to identify areas where elevated thermal stress, prolonged exposure, and demographic vulnerability overlap and to prioritize these areas for monitoring, preventive measures, and heat-mitigation and adaptation planning. The results demonstrate that a supervised ML approach, when combined with spatially harmonized reanalysis measures from open-access databases (e.g., ERA5-LAND, ERA5-HEAT, etc.), can estimate outdoor radiative dynamics with high accuracy while maintaining computational scalability and allowing real-time applicability. Indeed, the MLP model achieved high predictive performance during both validation and testing: the training and validation on the 2023 dataset yielded R^2^ = 0.99, RMSE = 1.4 °C, and MAE = 1.1 °C, while it reached R^2^ = 0.99, with RMSE = 2.7 °C and MAE = 1.9 °C when tested on a new dataset referring to the same real-world urban setting but in a different time period (Figure 7 and Figure 8). The preservation of R^2^ = 0.99 across both evaluation phases indicates that the model learned nonlinear relationships between meteorological data, radiative variables, surface descriptors, and solar geometry, rather than overfitting seasonal statistical patterns. In the context of the existing literature, these results are highly competitive. Jia et al. [12], in a comparative analysis across diverse outdoor environments, reported deep neural network performance with R^2^ around 0.88 and RMSE values in the range of approximately 1.9–2.0 °C, with site-specific deviations reaching up to 5 °C in complex urban canyons. Compared with those findings, the present study achieves higher explained variance (R^2^ = 0.99) and comparable RMSE during training. It is important, however, to interpret this comparison carefully. Jia et al. [12] relied on extensive and demanding in situ radiometric measurements at pedestrian scale in heterogeneous outdoor settings. The present study, by contrast, operates at the spatial resolution of ERA5-Land and ERA5-HEAT reanalysis products, which inherently smooth microscale heterogeneity. Therefore, while the achieved statistical performance is superior in terms of R^2^, the modeling scale and reference targets differ. The high performance observed here confirms that, at metropolitan resolution, ML can effectively estimate reanalysis-based t_r_ fields with fidelity. When compared to the work of Xie et al. [19], who reported RMSE values as low as approximately 0.49 °C using optimized neural networks, methodological differences become even more relevant. Xie et al. [19] trained their models on numerically simulated t_r_ fields obtained through heat balance equations in controlled urban configurations. Previous studies reported low RMSE values because neural networks were trained on simplified simulated data. In this work, reanalysis-derived t_r_ data collected over a complex urban area are used. For this reason, the slightly higher RMSE values (1.4 °C and 2.7 °C) are considered reasonable and consistent with real-world conditions. From an operational perspective, errors within this magnitude remain below thresholds that would alter stress-category classification in most UTCI-based applications. An essential aspect of the proposed methodology concerns the propagation of t_r_ estimation uncertainty into UTCI computation. Since UTCI is derived from a nonlinear regression approximation of the Fiala multi-node thermophysiological model, inaccuracies in t_r_ could theoretically amplify during physiological stress calculation. Nevertheless, UTCI performance remained stable. During training, UTCI estimation achieved R^2^ = 0.99, RMSE = 1.6 °C, and MAE = 1.2 °C, while independent August 2024 testing yielded R^2^ = 0.99, RMSE = 1.2 °C, and MAE = 0.9 °C. From a practical standpoint, uncertainties of this magnitude do not affect the assignment of thermal stress categories (e.g., moderate, strong, or very strong heat stress), which is the most relevant information for operational heat-risk management. From an operational perspective, the Monte Carlo analysis showed a generally limited propagation of uncertainty throughout the modeling chain. Among the individual MLP predictors, high-vegetation LAI produced the largest propagated variability in t_r_ (0.42 °C), followed by relative humidity and low-vegetation LAI (0.39 °C for both), whereas solar geometry variables had only a marginal influence. When all uncertain input variables were perturbed simultaneously, the predicted t_r_ showed a mean standard deviation of 0.88 °C, indicating that the MLP remained relatively stable within the adopted input-uncertainty ranges. In the subsequent analysis, the prediction uncertainty associated with the MLP-estimated t_r_ was propagated through the thermal-risk calculation chain (i.e., UTCI and UHRI computation). Using a coverage factor k = 2, the resulting propagated uncertainties U(y) were 0.67 °C for UTCI, 0.34 h for persistence, and 0.017 for UHRI. Overall, these results indicate that the framework maintains a relatively stable response to the considered uncertainty sources, while the uncertainty originating from the t_r_ prediction stage has a comparatively limited effect on the final normalized UHRI. Although the Monte Carlo analysis quantified uncertainty propagation in the continuous outputs of the proposed framework, the potential influence of UTCI uncertainty on discrete heat stress classification should also be considered. In particular, UTCI estimates within ±0.67 °C of the predefined classification thresholds (e.g., 26 °C, 32 °C, and 38 °C) may have uncertainty intervals extending into adjacent heat stress categories. For example, UTCI estimates between 31.33 °C and 32.67 °C may cross the 32 °C threshold separating moderate and strong heat stress when the reported uncertainty is considered. In the present study, the uncertainty analysis focused on UTCI and UHRI outputs, without explicitly quantifying the transition probabilities between discrete heat stress categories. Future developments could extend the proposed Monte Carlo framework to assess classification robustness near critical UTCI thresholds, thereby further supporting uncertainty-aware decision-making.

A further key contribution lies in the integration of thermal exposure measures through UTCI with socio-demographic vulnerability through the Urban Heat Risk Index (UHRI). By combining normalized UTCI intensity, heat stress persistence, and the proportion of residents aged 65 years, the framework translates climatic information into a spatially explicit risk metric. The Barcelona case study reveals a consistent spatial overlap between areas experiencing persistent strong heat stress and neighborhoods characterized by higher aging ratios, particularly within dense central districts. This convergence aligns with epidemiological evidence indicating elevated heat-related health risks among elderly populations and underscores the importance of coupling physiologically based indices with social vulnerability indicators. Although the vulnerability component is intentionally limited to a single demographic variable, the modular design allows for its future extension to multi-indicator vulnerability frameworks. The use of Over65 as the sole vulnerability proxy may introduce a representation bias in the current UHRI. Areas with a larger elderly population may receive greater vulnerability scores even where adaptive capacity is relatively high, whereas areas characterized by younger populations but substantial socioeconomic deprivation, poor housing conditions, limited access to cooling, social isolation, or energy poverty may be comparatively underrepresented. Therefore, the current vulnerability component should be interpreted specifically as an age-related indicator rather than as a comprehensive representation of social vulnerability. This limitation motivates the planned extension of the UHRI toward a multidimensional vulnerability component. Future developments should therefore incorporate additional socio-environmental determinants (e.g., income, housing conditions, access to cooling, social isolation, energy poverty, etc.) and explore data-driven calibration of weights against observed health outcomes where available. Another aspect to be addressed in future developments is the external validation of the UHRI against observed population-level health outcomes. In the present study, the UHRI is intended to provide a representation of spatial differences in combined outdoor thermal comfort, its persistence, and demographic vulnerability, rather than a direct quantitative estimate of heat-related morbidity or mortality. When sufficiently detailed local health datasets become available, future studies should compare UHRI values and risk classes with independent health-related indicators.

A relevant methodological aspect concerns the use of ERA5-HEAT as the reference t_r_ target instead of dense in situ radiometric measurements. This choice is consistent with the operational objective of the framework, which aims to provide spatially continuous and real-time thermal comfort and heat risk information at urban scale. ERA5-HEAT offers a standardized and temporally continuous reference [28,31], allowing the MLP to be trained over extended periods and subsequently used as a computationally efficient surrogate for hourly t_r_, UTCI, and UHRI estimation without requiring a dense radiometric network during deployment. This approach represents a trade-off between microscale detail and the spatial coverage, temporal continuity, and scalability required for operational applications. Accordingly, the reported agreement (R^2^ = 0.99) should be interpreted at the adopted reanalysis scale rather than as direct validation of pedestrian-scale t_r_ in complex urban environments. Fine-scale effects such as local shading, street geometry, and multiple reflections may not be fully represented and may consequently affect downstream UTCI and UHRI. Hence, future work will therefore complement the present urban-scale validation with targeted in situ radiometric measurements across different urban morphologies.

From an operational perspective, the next development step will be the implementation and pilot testing of the framework as a decision-support tool for municipalities and local authorities. The prototype will ingest updated meteorological data, estimate t_r_, calculate UTCI and update UHRI maps in real time. The interface will provide georeferenced thermal comfort and risk maps, prioritization of administrative areas according to UHRI and thermal-stress severity, and early-warning recommendations when predefined thresholds are reached or exceeded. The tool is intended to support both short-term and long-term decisions. During heat events, it can help local authorities identify areas requiring closer monitoring, timely communication, and preventive measures. Over longer time scales, recurrent thermal-stress and UHRI hotspots can support the prioritization of urban heat-mitigation and adaptation interventions. The framework is therefore suited to city- and district-scale screening, continuous monitoring, early-warning support, and spatial prioritization, but it is not intended to replace detailed pedestrian-scale microclimatic assessments or to act as the sole criterion for public-health emergency interventions. Future developments will include pilot testing in Barcelona, targeted in situ validation, comparison with local health data where available, extension of the vulnerability component, and testing in other cities and climatic contexts.

Overall, the proposed framework provides a scalable, operational, and transferable approach to translate urban heat exposure and social vulnerability into actionable risk information, supporting evidence-based planning and more resilient heat-management strategies in metropolitan contexts.

## Figures and Tables

**Figure 1:**
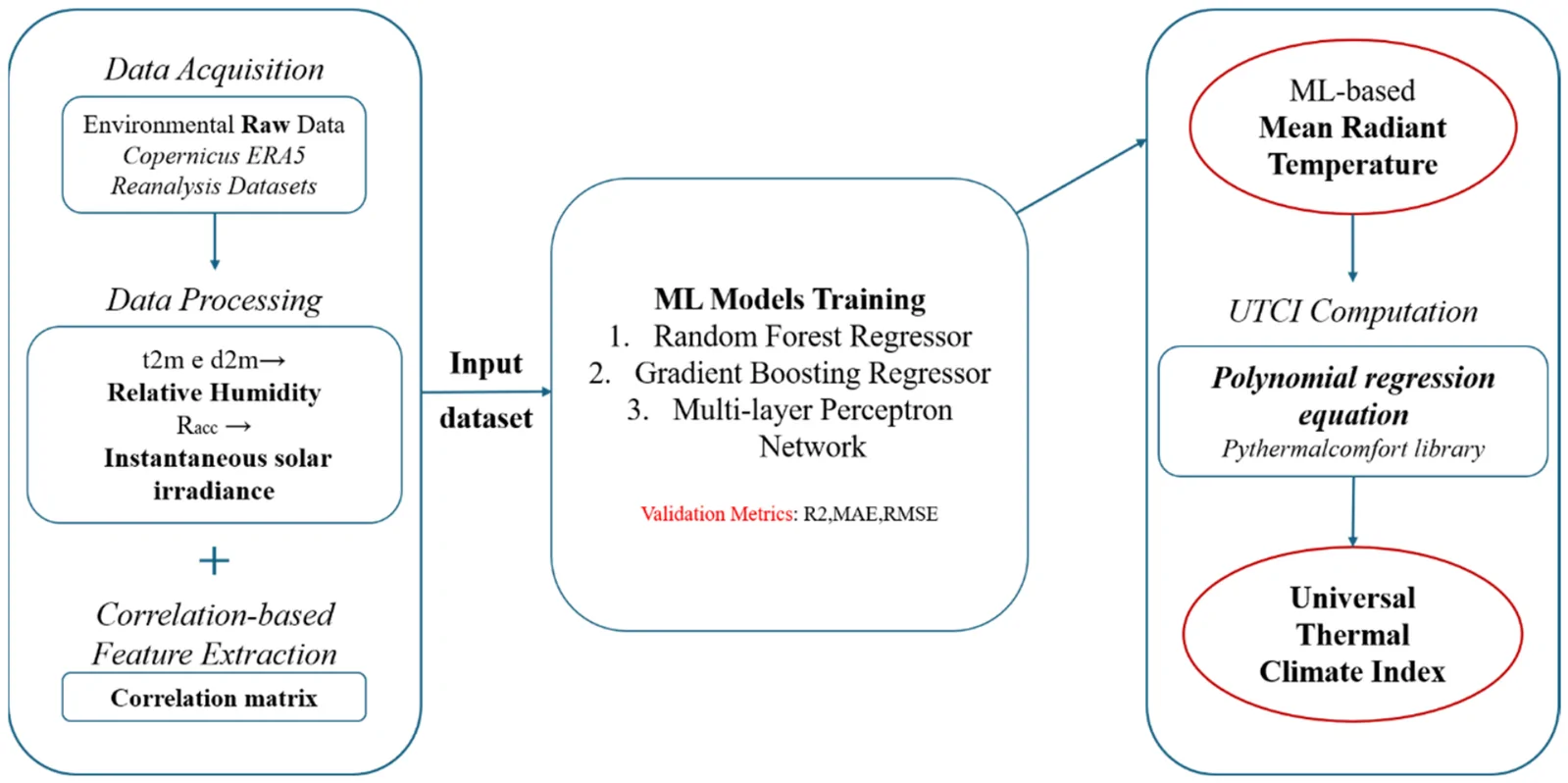
ML-based measurement workflow proposed in this paper.

**Figure 2:**
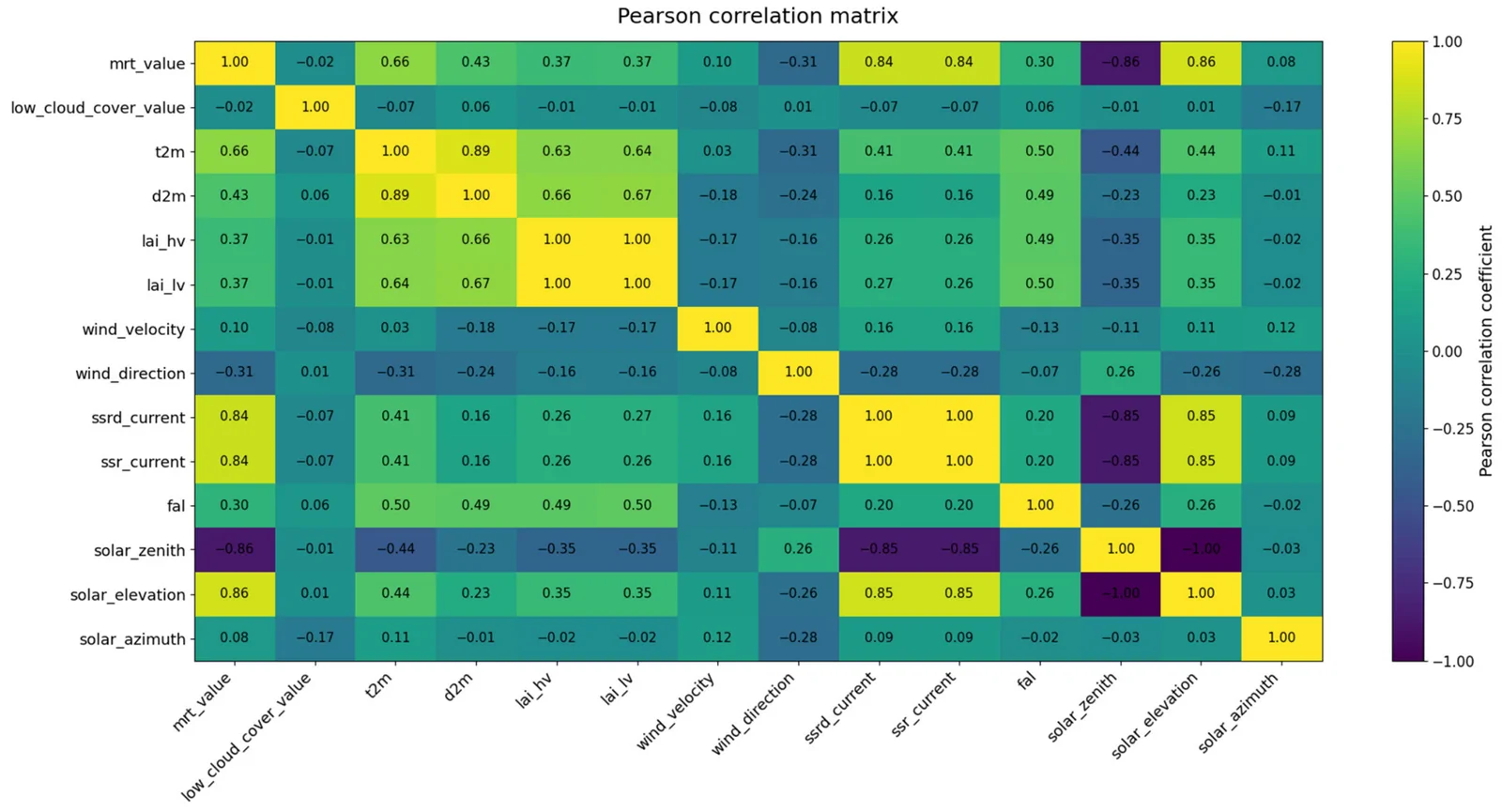
Pearson correlation matrix of the candidate meteorological, radiative, environmental, and solar geometry variables considered for MLP development.

**Figure 3:**
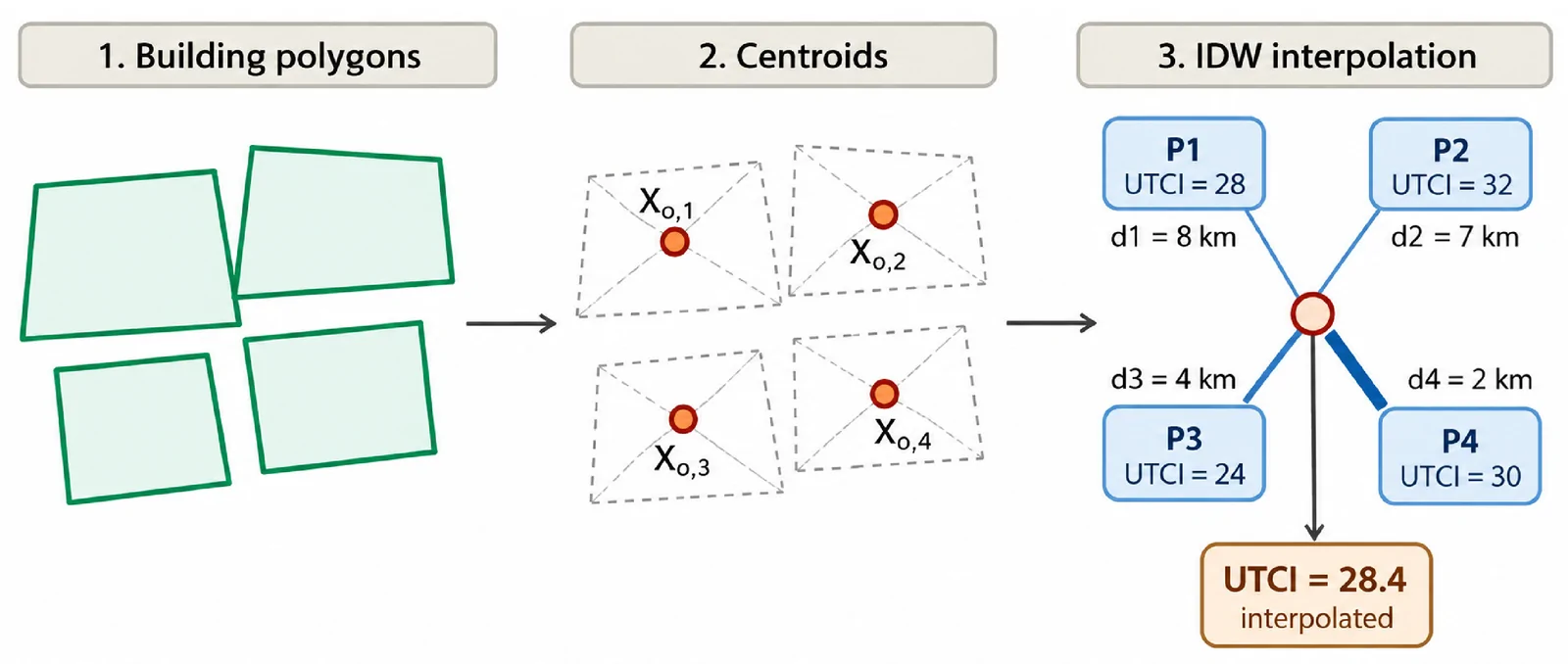
Schematic representation of the IDW-based workflow applied for spatial aggregation and estimation of UTCI values at administrative-unit scale. Green elements represent the building polygons, orange circles indicate centroid/interpolation points, blue elements represent the surrounding UTCI input points, and the brown box indicates the resulting interpolated UTCI value.

**Figure 4:**
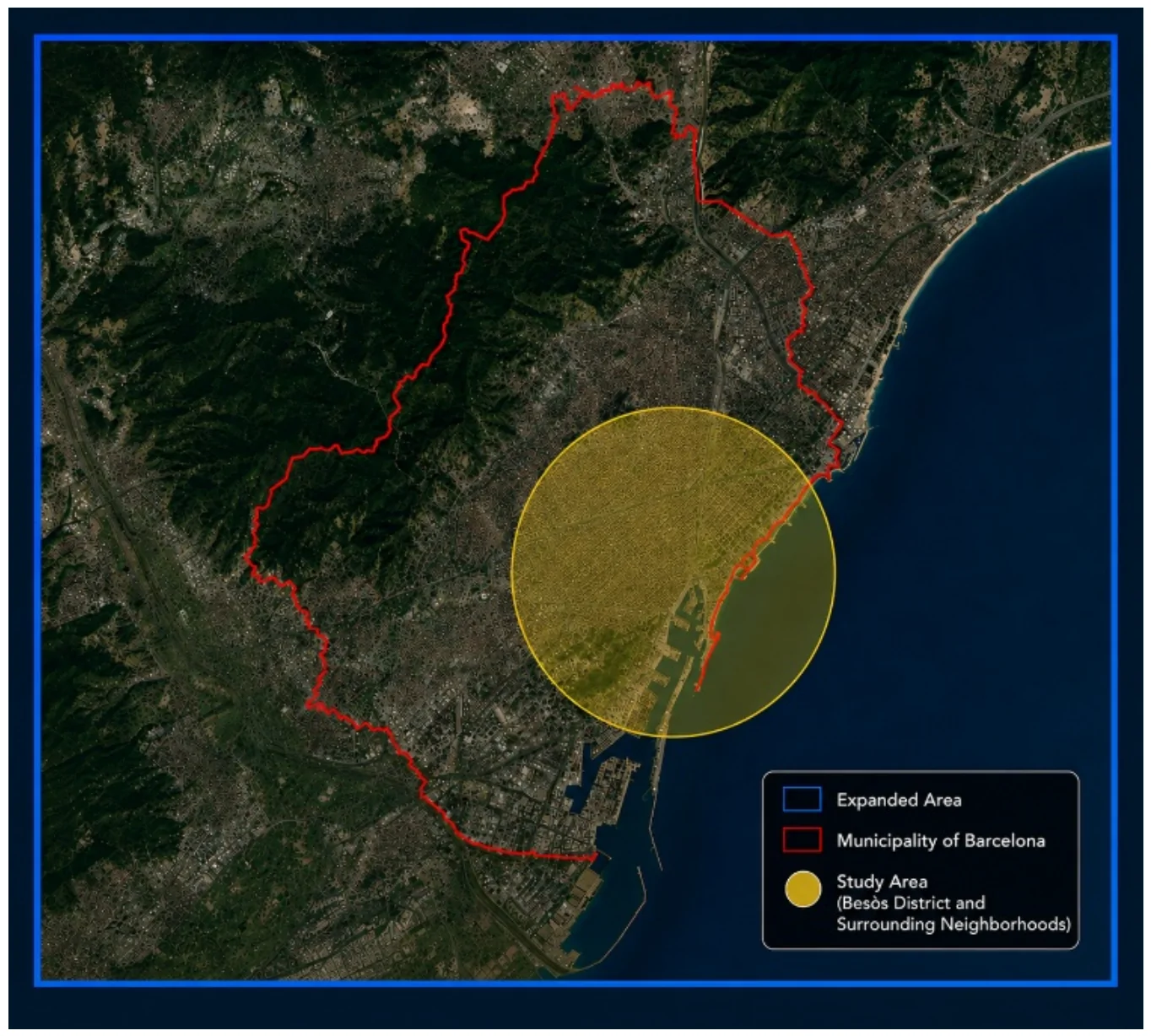
Area under study: the red line outlines the municipality of Barcelona, and the yellow circle covers the San Martì district and surrounding neighborhoods.

**Figure 5:**
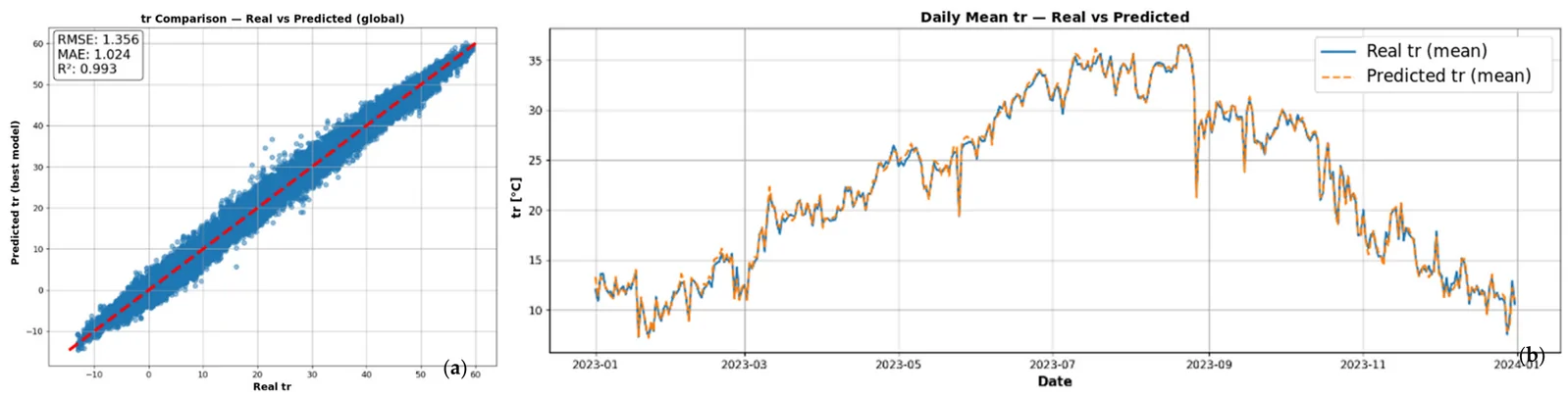
(**a**) Correlation results between the ERA-HEAT t_r_ and predicted t_r_. (**b**) Temporal comparison between predicted and ERA5-HEAT t_r_.

**Figure 6:**
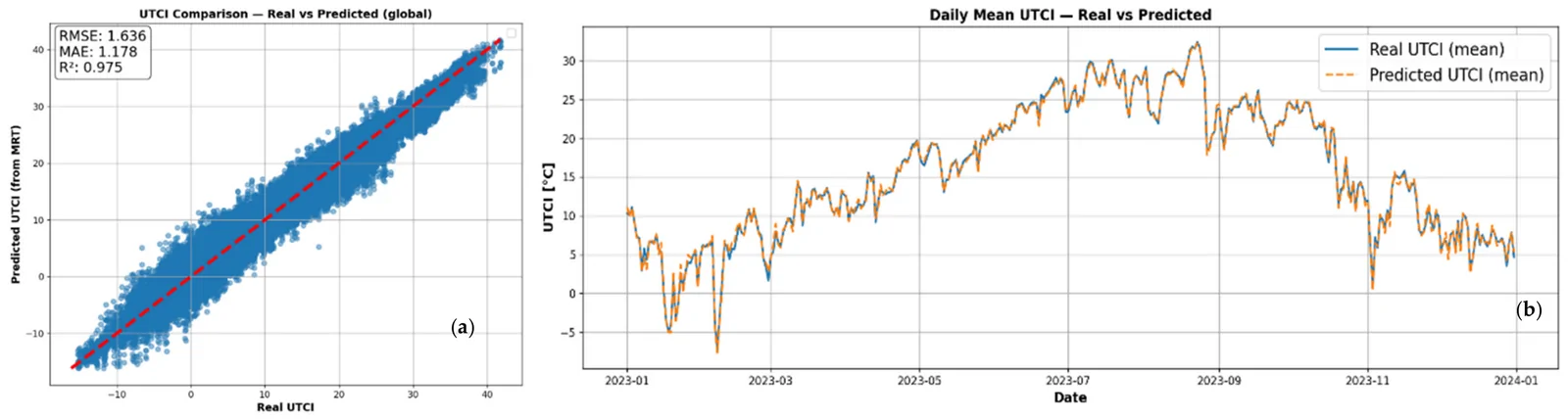
(**a**) Correlation results between ERA-HEAT UTCI and predicted UTCI. (**b**) Temporal comparison between predicted and ERA5-HEAT UTCI.

**Figure 7:**
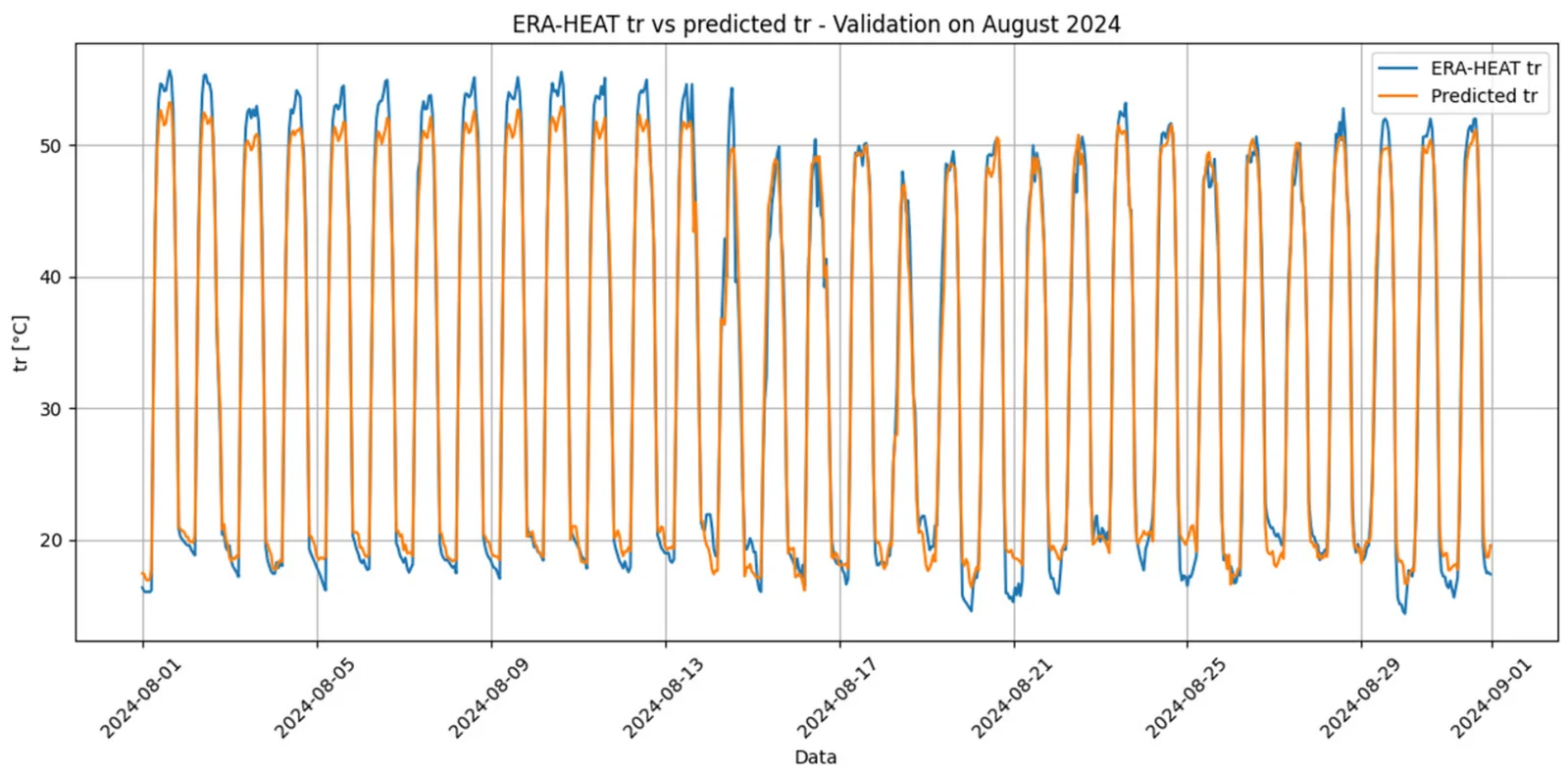
Comparison between estimated and ERA5-HEAT t_r_ values for August 2024 (testing dataset). The data represent daily mean t_r_ values.

**Figure 8:**
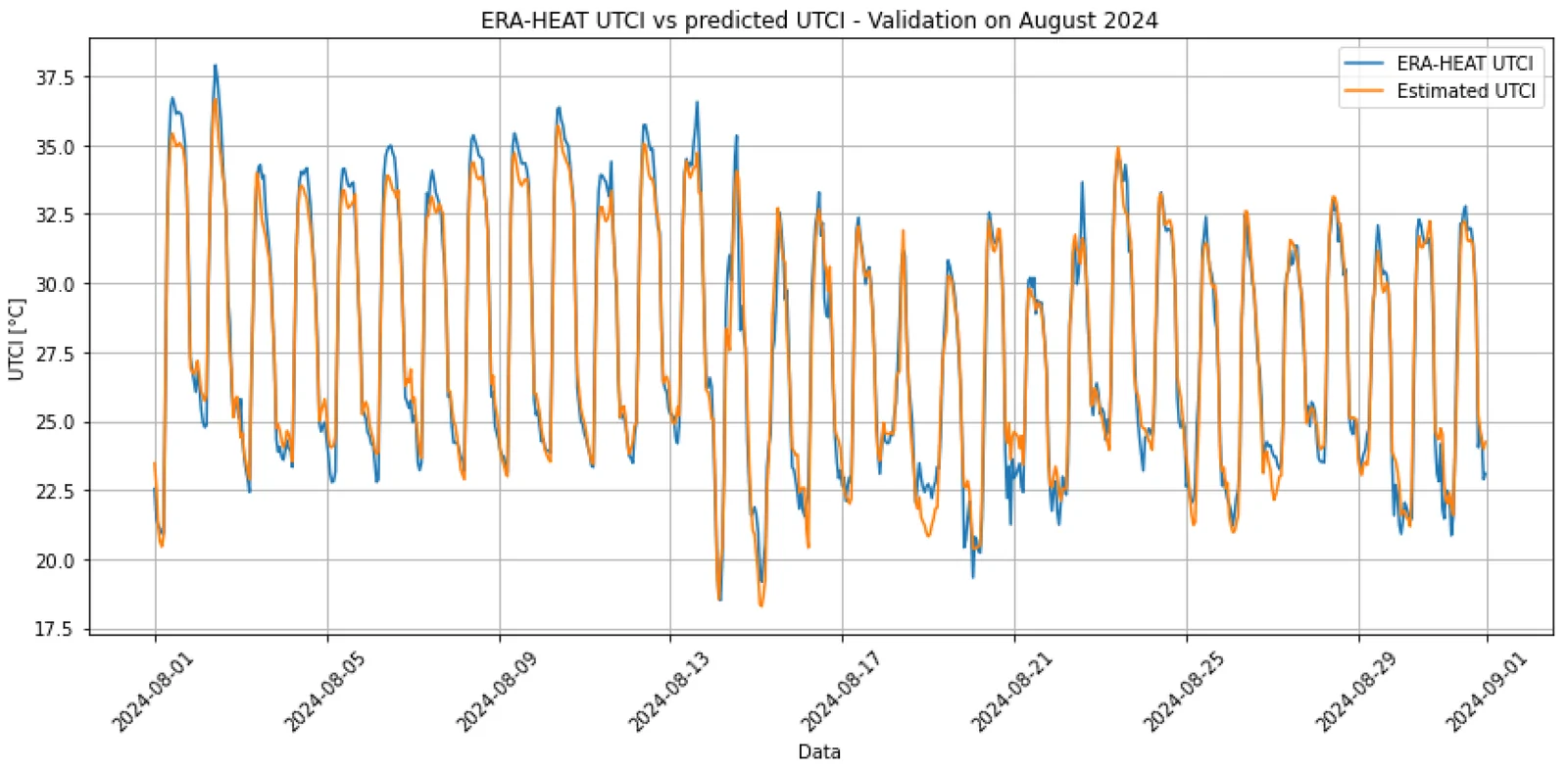
Comparison between actual and calculated UTCI for August 2024 (testing dataset). The data represent daily mean UTCI values.

**Figure 9:**
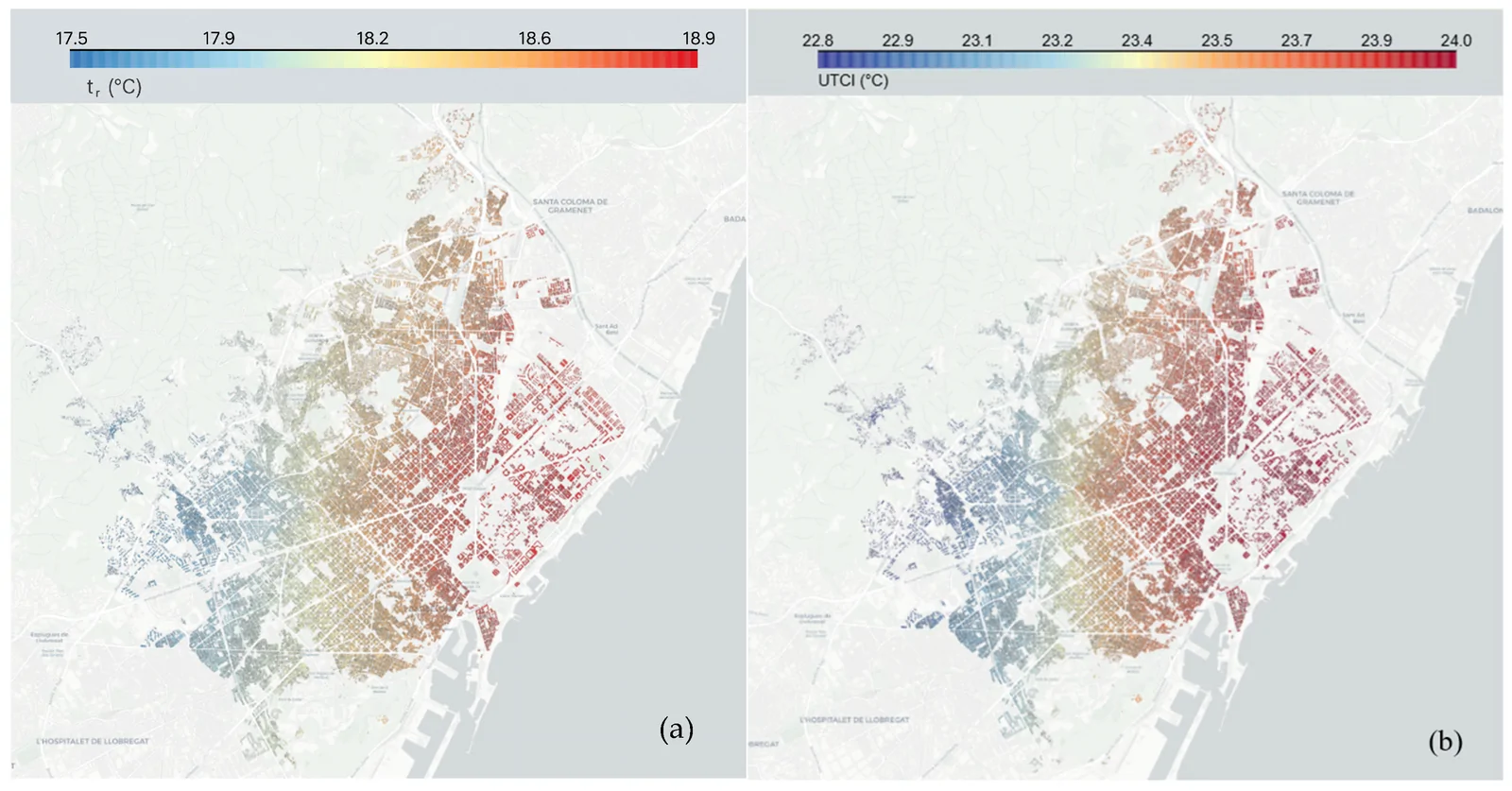
(**a**) Spatial distribution of the monthly mean t_r_ at 01:00, obtained by averaging all t_r_ values at 01:00 recorded during August 2024. (**b**) Corresponding monthly mean UTCI at 01:00, computed from the mentioned t_r_.

**Figure 10:**
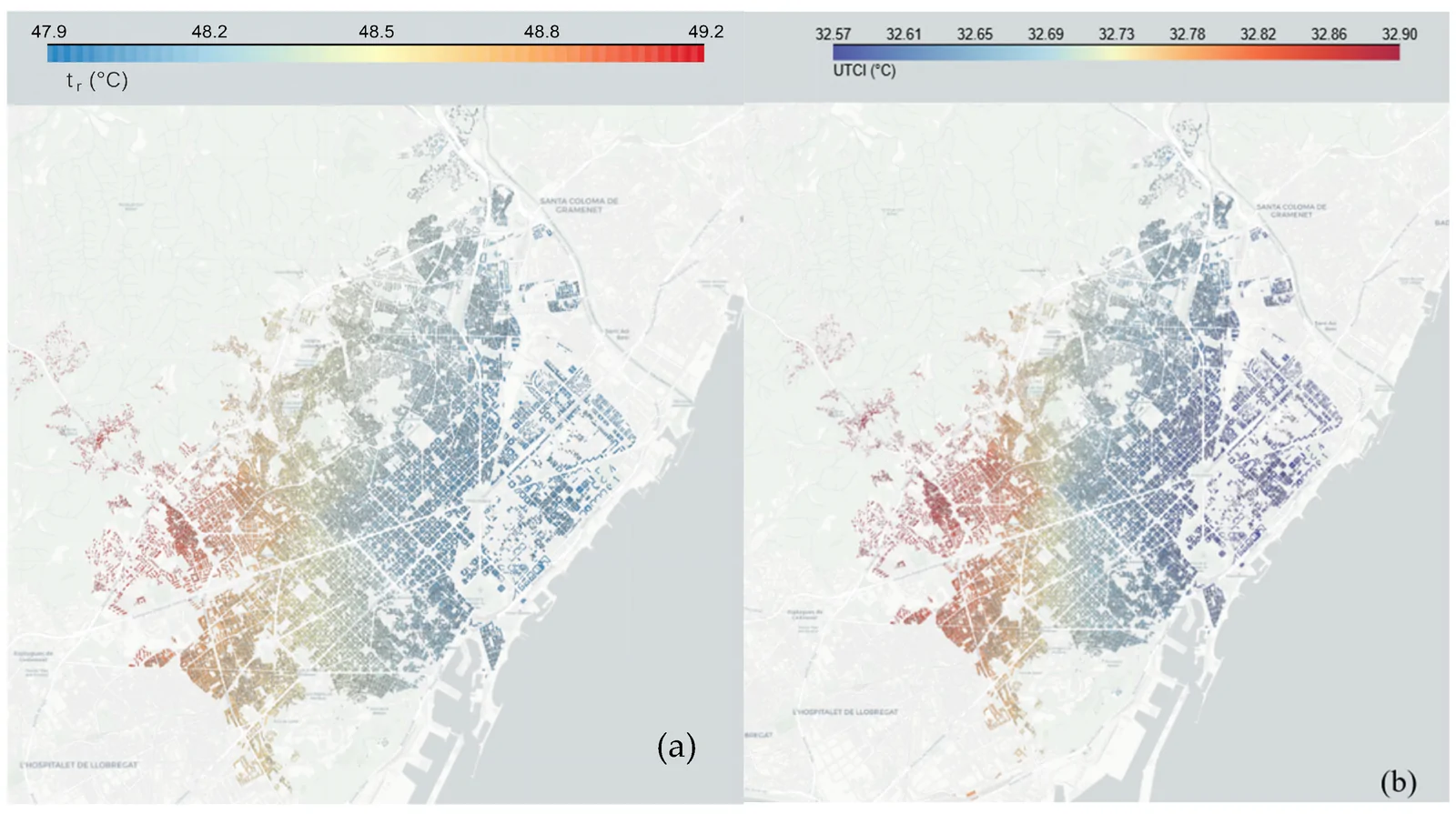
(**a**) Spatial distribution of the monthly mean t_r_ at 09:00, obtained by averaging all t_r_ values at 09:00 recorded during August 2024. (**b**) Corresponding monthly mean UTCI at 09:00, computed from the mentioned t_r_.

**Figure 11:**
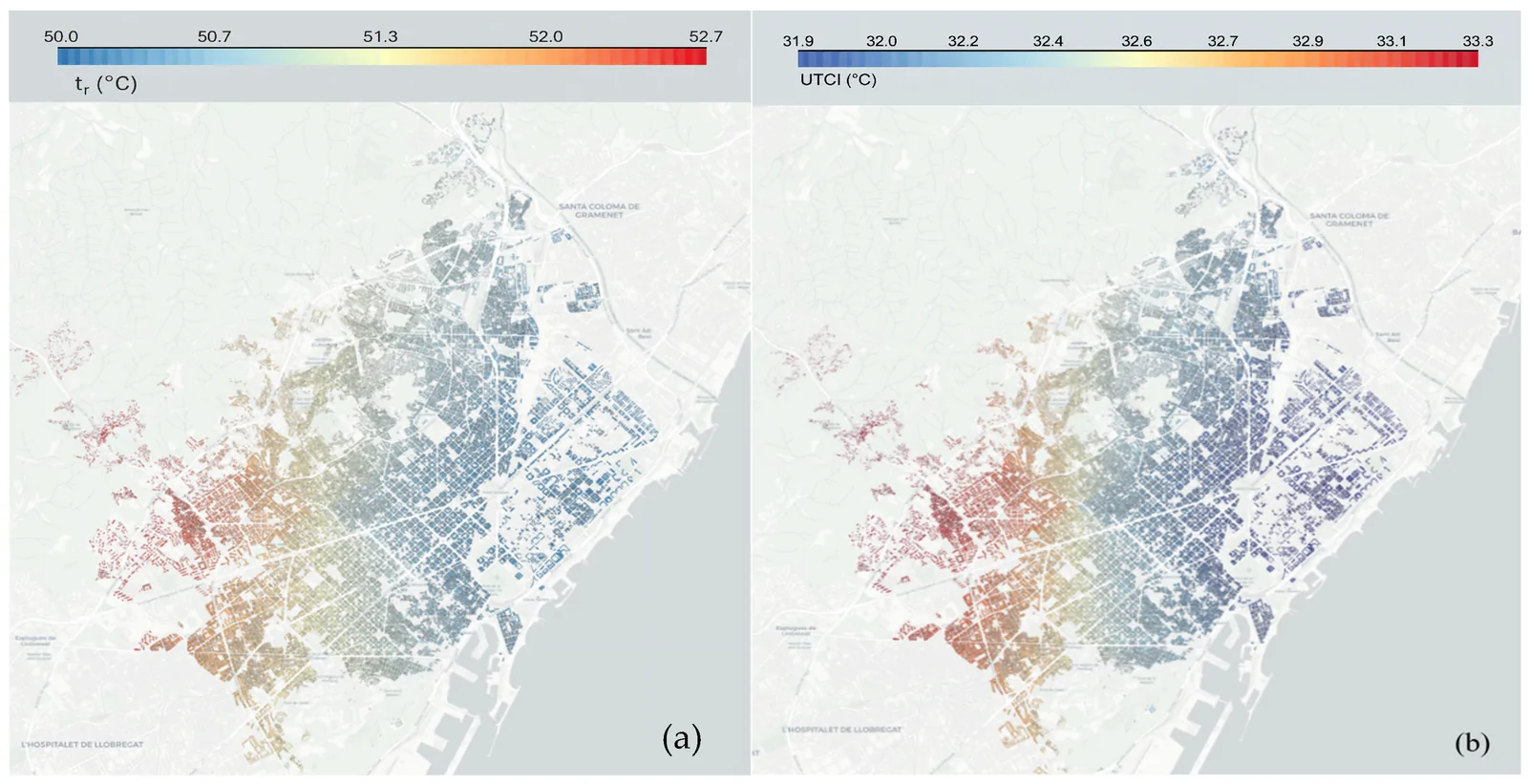
(**a**) Spatial distribution of the monthly mean t_r_ at 13:00, obtained by averaging all t_r_ values at 13:00 recorded during August 2024. (**b**) Corresponding monthly mean UTCI at 13:00, computed from the mentioned t_r_.

**Figure 12:**
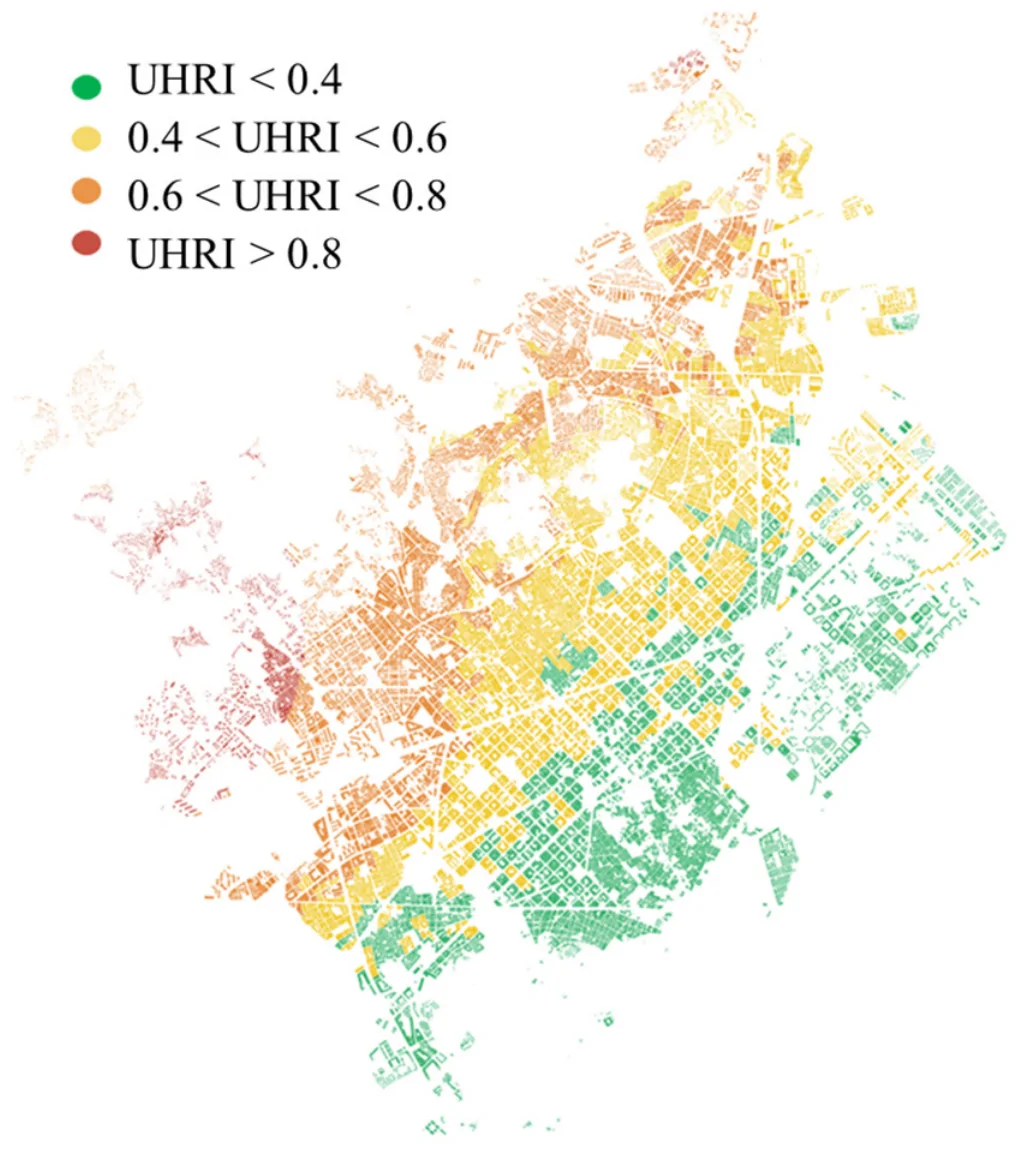
Resulting risk map based on preliminary risk score calculation.

**Figure 13:**
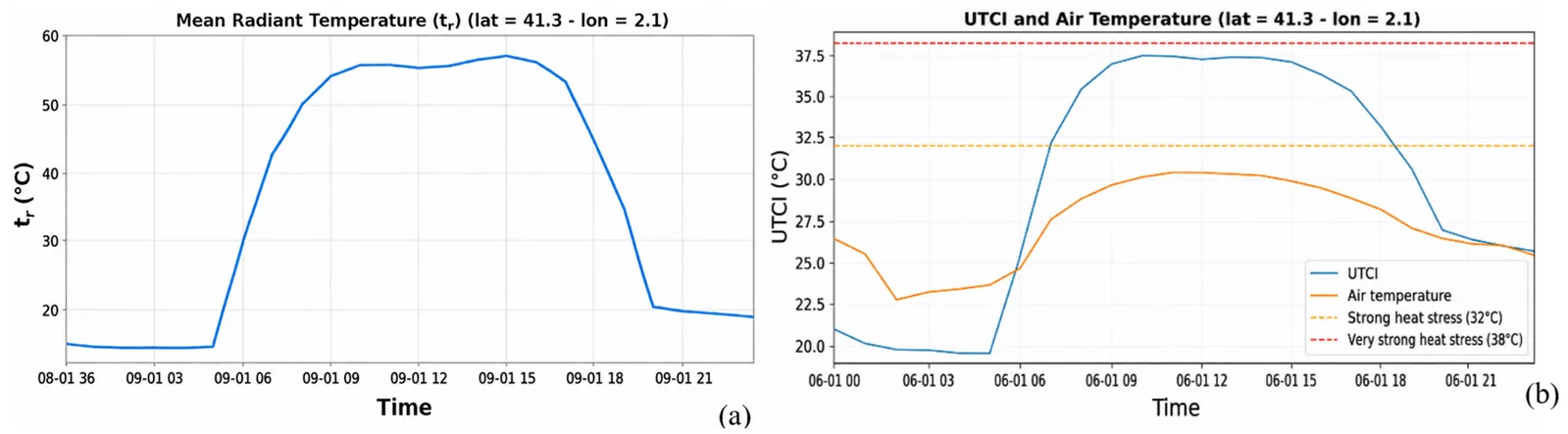
(**a**) Example of real-time t_r_ measurement applied in the district under study. (**b**) Related real-time measurement of UTCI with operational ranges (32 °C, 38 °C).

**Table 1:** UTCI scale and related heat stress level established by the International Society of Biometeorology (ISB) [12].

UTCI (°C)	Stress Category
UTCI > 46	Extreme heat stress
38 < UTCI < 46	Very strong heat stress
32 < UTCI < 38	Strong heat stress
26 < UTCI < 32	Moderate heat stress
9 < UTCI < 26	No heat stress
0 < UTCI < 9	Slight cold stress
−13 < UTCI < 0	Moderate cold stress
−27 < UTCI < −13	Strong cold stress
−40 < UTCI < −27	Very strong cold stress
UTCI < −40	Extreme cold stress

**Table 2:** List of abbreviations used in the current work.

**AI**	Artificial Intelligence	**GBR**	Gradient Boosting Regressor
**ANN**	Artificial Neural Network	**LAI**	Leaf Area Index
**API**	Application Programming Interface	**LST**	Land Surface Temperature
**CFS**	Correlation-based Feature Selection	**MAE**	Mean Absolute Error
**DB**	Database	**MAPE**	Mean Absolute Percentage Error
**DNN**	Deep Neural Network	**ML**	Machine Learning
**ECMWF**	European Centre for Medium-Range Weather Forecasts	**MLP**	Multi-Layer Perceptron
**ERA5**	Fifth-generation ECMWF Atmospheric Reanalysis	**NRMSE**	Normalized Root Mean Squared Error
**ERA5-HEAT**	ERA5 Human Thermal Comfort Reanalysis	**PET**	Physiological Equivalent Temperature
**ERA5-Land**	ERA5 Land Reanalysis	**RFR**	Random Forest Regressor
**FAL**	Forecast Albedo	**RH**	Relative Humidity
**HVI**	Heat Vulnerability Index	**RMSE**	Root Mean Squared Error
**HVAC**	Heating, Ventilation and Air Conditioning	**SSP**	Shared Socioeconomic Pathway
**IDW**	Inverse Distance Weighting	**UHI**	Urban Heat Island
**IPCC**	Intergovernmental Panel on Climate Change	**UHRI**	Urban Heat Risk Index
**ISB**	International Society of Biometeorology	**UTCI**	Universal Thermal Climate Index
**tr**	Mean Radiant Temperature	**Racc**	Accumulated Shortwave Solar Radiation
**Ta**	Indoor temperature	**R2**	Coefficient of determination
**T2m**	Outdoor temperature at 2 m	**Td**	Dew-Point temperature

**Table 3:** Input features and target of the ML-based model approach.

Input features	Solar radiation (R_acc_, W/m^2^), Albedo (FAL, u.a.), Leaf Area Index (LAI, u.a.), Wind speed and direction (u_wind_, d_wind,_ m/s), Air temperature (t2m °C), Relative humidity (RH, %), Cloud coverage (u.a.), Solar azimuth (°), Zenith angles (°)
Data Source	ERA5-LAND, ERA-HEAT, ERA5
Target	Mean Radiant Temperature (°C)
Data Source	ERA-5HEAT

**Table 4:** ML model architectures and related hyperparameters.

MLP	GB	RFR
Size of Hidden layers = (128, 64) Activation function = relu Alpha = 1 × 10^−3^ Max iteration = 500 Random state = 42	N. estimators = 300 Learning rate = 0.05 Max depth = 4	N. estimators = 50 Max depth = 10 Min samples split = 5 Min samples leaf = 2

**Table 5:** Validation metrics obtained during model training using the 2023 dataset for the selected ML models.

	R^2^	RMSE	MAE	NRMSE
MLP	0.99	1.4 °C	1.1 °C	1.9%
GB	0.99	1.8 °C	1.4 °C	2.5%
RFR	0.99	1.9 °C	1.4 °C	2.6%

**Table 6:** UHRI ranges and related risk.

***Low***	UHRI < 0.40
***Moderate***	0.40 ≤ UHRI < 0.60
***High***	0.60 ≤ UHRI < 0.80
***Very high***	UHRI ≥ 0.80

**Table 7:** Input features and data source for the real-time application.

Input Features	Data Source
Solar radiation (W/m^2^), R_acc_	Open-Meteo API
Wind speed and direction (m/s), u_wind_, d_wind_	Open-Meteo API
Air temperature (°C), t2m	Open-Meteo API
Relative humidity (%), RH	Open-Meteo API
Cloud coverage (u.a.)	Open-Meteo API
Solar azimuth (°C)	Computed using PVlib library
Zenith angles (°C)	Computed using PVlib library
Albedo (u.a.), FAL	ERA5 Land
Leaf Area Index (u.a.), LAI	ERA5 Land
Solar radiation (W/m^2^), R_acc_	Open-Meteo API

**Table 8:** Validation metrics of the MLP model for t_r_ prediction and corresponding UTCI-related metrics. Training and validation were performed using the complete 2023 dataset, while testing was conducted using data from August 2024.

	Training and Validation		Testing	
MLP	MAE	1.1 °C	MAE	1.9 °C
	RMSE	1.4 °C	RMSE	2.6 °C
	R^2^	0.99	R^2^	0.99
UTCI	MAE	1.2 °C	MAE	0.9 °C
	RMSE	1.6 °C	RMSE	1.2 °C
	R^2^	0.99	R^2^	0.99

**Table 9:** Input uncertainty values adopted in the Monte Carlo analysis and the resulting standard deviation of the predicted t_r_ with the sensitivity coefficient for the one-at-a-time perturbation of each variable.

Input Parameters	Input Uncertainty, *σ_i_*	t_r_ Propagated Standard Uncertainty	Sensitivity Coefficient
Solar radiation (R_acc_, W/m^2^)	9.91 W/m^2^	0.29 °C	0.029 °C/(W/m^2^)
Albedo (FAL, u.a.)	0.001	0.12 °C	120 °C/FAL unit
Leaf Area Index high (LAI, u.a.)	0.05	0.42 °C	8.40 °C/LAI unit
Leaf Area Index low (LAI, u.a.)	0.05	0.39 °C	7.80 °C/LAI unit
Wind speed (u_wind_,_,_ m/s)	0.31	0.11 °C	0.355 °C/(m/s)
Direction speed (d_wind_, °)	11.12°	0.093 °C	0.0084 °C/°
Air temperature (t2m, °C)	0.28 °C	0.078 °C	0.279
Relative humidity (RH, %)	1.80 %	0.39 °C	0.217 °C
Cloud coverage (u.a.)	0.064	0.18 °C	2.81 °C/unit
Solar azimuth (°)	0.1°	0.0025 °C	0.025 °C/°
Zenith angles (°)	0.1°	0.018 °C	0.180 °C/°

**Table 10:** Propagation of MLP-predicted t_r_ uncertainty through UTCI, persistence, and UHRI, including parameter estimates, propagated uncertainty U(y) with coverage factor k = 2, and sensitivity coefficients.

Uncertainty Source	t_r_ (°C)	UTCI (°C)	UTCI (°C), Persistence (h)
Standard uncertainty	±2.6 °C	±0.67 °C	±0.67 °C, 0.34 h
Measured variable	UTCI (°C)	Persistence (h)	UHRI
Parameter Estimate	27.8 °C	1.1 h	0.33
Uncertainty, U(y) (k = 2)	U_UTCI_ = 0.67 °C	U_persistence_ = 0.34 h	U_UHRI_ = 0.017
Sensitivity coefficient	0.26	0.51	--

## Data Availability

The data presented in this study are available on request from the corresponding author.
